# Microplastics-Mediated Behavior of Potentially Toxic Elements in Plant–Soil Systems: Adsorption, Bioavailability, and Phytotoxicity

**DOI:** 10.3390/toxics14080730

**Published:** 2026-08-17

**Authors:** Shaohong You, Kaiyang Ying, Songhao Zhang, Caixing Lai, Habib Ullah, Ahmed Mahmoud Ismail, Guo Yu

**Affiliations:** 1 Guangxi Key Laboratory of Environmental Pollution Control Theory and Technology, Guilin University of Technology, Guilin 541006, China; youshaohong@glut.edu.cn (S.Y.); naduo339@163.com (K.Y.); 13051105577@163.com (S.Z.); 2 Guangxi Key Laboratory of Green Preparation and Application of Inorganic Materials, Guangxi Science & Technology Normal University, Laibin 546199, China; 3 School of Intelligent Electrical and New Energy, Qingdao Hengxing University of Science and Technology, Qingdao 266042, China; habib901@zju.edu.cn; 4 Innovation Center of Yangtze River Delta, Zhejiang University, Jiashan 314100, China; 5 Pests and Plant Diseases Unit, College of Agricultural and Food Sciences, King Faisal University, Al-Ahsa 31982, Saudi Arabia; amismail@kfu.edu.sa; 6 Center for Water and Ecology, State Key Laboratory of Iron and Steel Industry Environmental Protection, School of Environment, Tsinghua University, Beijing 100084, China; 7 University Engineering Research Center of Watershed Protection and Green Development, Guilin University of Technology, Guilin 541006, China; 8 Key Laboratory of Carbon Emission and Pollutant Collaborative Control, Education Department of Guangxi Zhuang Autonomous Region, Guilin University of Technology, Guilin 541006, China

**Keywords:** microplastics, potentially toxic elements, trace metals, metalloids, adsorption, desorption, bioavailability, eco-corona

## Abstract

Microplastics (MPs) and potentially toxic elements (PTEs) increasingly co-occur in agricultural and peri-urban soils, yet their combined effects on adsorption, mobility, bioavailability, and phytotoxicity are highly context-dependent. This review synthesizes plant–soil evidence by focusing on the interacting roles of MP polymer type, particle size and shape, aging/weathering state, soil geochemistry, dissolved organic matter, and rhizosphere processes. Across the reported studies, MP-PTE interactions show several major directions of changes: MPs may reduce PTE lability by promoting adsorption, aggregation, or sequestration within coated surfaces and soil aggregates; conversely, they may increase PTE mobility and plant exposure when reversible binding, dissolved organic ligands, pH shifts, or particle transport deliver labile PTEs to root-active zones. Dose-dependent and biphasic responses are also common, with low MP additions sometimes attenuating stress while higher doses intensify toxicity. Quantitatively, available crop studies show that intensified co-exposure can reduce plant biomass by approximately 10.2–29.3%, depending on crop species, plant organ, MP type, dose, and PTE identity, whereas antagonistic or neutral responses are also reported under other exposure conditions. The strongest evidence currently exists for Cd and As, but this review also considers Pb, Cu, Zn, Ni, Cr, and Hg to represent chemically distinct cationic, metalloid, and redox-sensitive PTEs. Overall, MPs should not be treated only as passive contaminant carriers; they act as dynamic reactivity modifiers that can function as sinks, vectors, or indirect regulators of PTE bioavailability depending on soil and rhizosphere boundary conditions.

## 1. Introduction

The pervasive accumulation of plastic debris in terrestrial ecosystems has emerged as a global environmental concern, with microplastics (MPs; particles < 5 mm) receiving particular attention due to their persistence, mobility, and potential to act as vectors for co-occurring contaminants [1,2]. Concurrently, potentially toxic elements (PTEs)—including cadmium (Cd), lead (Pb), arsenic (As), copper (Cu), and zinc (Zn)—remain among the most hazardous soil pollutants, owing to their non-biodegradability, bioaccumulation tendency, and severe phytotoxic effects at trace concentrations [3,4]. Although MPs and PTEs have historically been investigated as separate stressors, mounting evidence indicates that their co-occurrence in agricultural soils—from plastic mulching, sewage sludge application, atmospheric deposition, and irrigation with contaminated water—modulates the environmental fate, bioavailability, and toxicity of PTEs in plant–soil systems [5,6,7].

The central paradigm underpinning current research is that MPs alter PTE behavior through three non-mutually exclusive mechanisms: (1) direct adsorption/desorption processes at the MP–water–soil interface; (2) modification of soil physicochemical and biogeochemical properties (e.g., pH, redox potential, dissolved organic matter, and microbial community structure); and (3) rhizosphere-mediated effects on root exudation, metal-transporter expression, and plant uptake kinetics [8,9]. However, the direction and magnitude of these effects are highly context-dependent, leading to contradictory reports. For instance, some studies demonstrate that polyethylene (PE) MPs increase Cd bioavailability by disrupting soil aggregates and reducing pH [10], whereas others show that polyamide (PA) or polylactic acid (PLA) MPs decrease Cd phytoavailability through enhanced sorption or stimulated microbial immobilization [11,12]. Such discrepancies highlight the urgent need to systematically unravel the controlling factors and underlying mechanisms governing MP–PTE interactions. In this context, MPs should not be viewed as inert particles but as active components of soil systems that function simultaneously as sorbents, mobile carriers, and modifiers of the soil microenvironment. At the same time, PTE behavior is governed by speciation, competition for sorption sites, redox state, dissolved organic matter, and root-induced chemical gradients. The resulting exposure of plants therefore depends on coupled processes rather than on total concentration alone [13,14,15].

Based on a critical evaluation of the available literature, this review identifies three recurring mechanistic domains through which MPs may influence PTE behavior in plant–soil systems. First, the physicochemical properties of MPs, including polymer type (e.g., biodegradable vs. conventional), particle size, morphology, surface charge, weathering degree, and specific surface area, regulate adsorption capacity, binding affinity, and desorption reversibility. Biodegradable MPs are considered separately from conventional polymers because their degradation products, additive release, surface oxidation, and microbial colonization may produce stronger rhizosphere-mediated effects on PTE mobility and bioavailability than relatively inert PE (polyethylene), PP (polypropylene), or PS (polystyrene) particles. Second, soil biogeochemical conditions, including pH, organic matter, clay minerals, Fe/Mn oxides, competing ions, and dissolved organic matter, determine whether MP-associated PTEs are immobilized, mobilized, or redistributed among labile and non-labile pools. Third, rhizosphere processes, including root exudation, microbial activity, biofilm formation, and plant-specific uptake traits, can modify PTE speciation and phytoavailability. These three domains are treated here as organizing themes emerging from the reviewed literature rather than as experimentally tested hypotheses. The framework proposed here does not assign quantitative weighting or universal hierarchical importance to individual mechanisms because the reviewed studies differ substantially in polymer type, particle size, soil properties, exposure level, plant species and analytical method. Instead, it identifies recurring interacting process domains reported across the literature and uses them as a conditional interpretive structure.

Although these mechanisms are repeatedly reported across the literature, the evidence remains fragmented and context-dependent. Existing reviews have addressed MP—PTE interactions from different perspectives, including adsorption behavior, soil contamination, plant uptake, and ecotoxicity; however, few have explicitly connected adsorption/desorption behavior with soil-mediated mobility, rhizosphere transformation, PTE bioavailability, and phytotoxic outcomes within a single plant–soil framework. Wang et al. (2020) provided a comprehensive overview of the adsorption mechanisms of PTEs onto MPs in aquatic environments but with limited emphasis on soil matrices or plant responses [16]. Khalid et al. (2020) focused on the combined toxicological effects of MPs and PTEs on soil biota and plants yet did not systematically disentangle the relative contributions of adsorption versus soil biogeochemical modifications [6]. Yu et al. (2022) reviewed the influence of MP aging on metal adsorption but largely excluded the rhizosphere as a dynamic interface [8]. More recently, Liu et al. (2024) systematically examined the interaction of microplastics with heavy metals in soil, covering mechanisms (surface complexation, electrostatic interaction, bioaccumulation, and precipitation), influencing factors (MP characteristics, HM properties, and soil conditions), and biological effects; however, this synthesis did not develop an integrated hypothesis-driven framework that unifies MP properties, soil biogeochemistry, and rhizosphere processes into a coherent predictive model for plant–soil systems [9]. Consequently, existing syntheses tend to treat the three control domains separately, leading to a fragmented understanding and an inability to reconcile contradictory findings.

Two further recent syntheses illustrate the same pattern. Bian et al. (2024) [17] critically reviewed MP-heavy metal co-pollution in agricultural soils, including formation mechanisms, spatial overlap of MP and metal contamination, and the “Trojan horse” co-toxicity concept, but did not extend the discussion to rhizosphere-specific processes or plant physiological responses. Xiong et al. (2024) [12] addressed the broader role of MPs as vectors for co-occurring pollutants but did not establish a dedicated plant–soil bioavailability-to-phytotoxicity framework. In contrast, the present review connects adsorption/desorption behavior, soil-mediated mobility, rhizosphere transformation, and phytotoxic outcomes within an explicit conditional framework (sink/vector/indirect modifier; Table 1) rather than reiterating MP–metal contact mechanisms or co-occurrence patterns already covered by previous reviews.

Against this background, the present review adopts a literature-grounded perspective that MP effects are jointly regulated by particle properties, soil biogeochemistry, and rhizosphere activity. Evidence indicates that polymer type, particle size, surface oxidation, soil pH, ionic strength, aggregation, and dissolved organic matter collectively influence sorption processes and plant exposure pathways [16,18,19]. The thematic structure used here represents a conditional interpretive model rather than a fully parametrized quantitative prediction tool. It was developed from gaps in existing reviews that commonly treat MP properties, soil processes, and rhizosphere interactions as independent domains. Rather than ranking individual factors such as root exudates, microbial biofilms, dissolved organic matter (DOM), pH, or mineral surfaces as universally dominant, this framework links three controlling groups—microplastic properties, soil biogeochemical conditions, and rhizosphere/plant traits—to four outcomes: adsorption/desorption, mobility, bioavailability, and phytotoxicity (Table 1).

Within this structure, microplastics are expected to behave mainly as sinks when sorption is strong and desorption is limited within stable aggregates or coated surfaces. They are expected to behave as vectors when particle transport, reversible binding, dissolved organic ligands, or root-zone delivery increases the flux of labile, potentially toxic elements. They are expected to act as indirect reactivity modifiers when they alter soil pH, dissolved organic matter, aggregation, microbial activity, or rhizosphere chemistry without directly transporting substantial contaminant mass. Building on these gaps, this review differs from prior work in three key respects. First, it explicitly evaluates the above thematic structure through systematic literature synthesis rather than merely cataloging observations. Second, it integrates adsorption science with soil biogeochemistry and plant physiology, advancing beyond the conventional vector/sink perspective to conceptualize MPs as reactivity modifiers that influence PTE speciation, lability, and plant availability via coupled mechanisms. Third, it proposes a unified thematic structure that links MP properties, soil conditions, and plant traits to predict PTE behavior, thereby identifying key knowledge gaps and providing testable directions for future research.

**Table 1:** Knowledge gaps and their possible solutions.

Previous Review Focus	Main Contribution	Remaining Limitation	Gap Addressed by This Review	References
MP adsorption of metals	Summarized sorption mechanism and polymer surface effects	Often emphasized aquatic or simplified batch systems	Links sorption/desorption to soil mobility and plant exposure	[20]
MP effects on soil organisms/plants	Reviewed toxicity and growth effects	Limited mechanistic connection to PTE lability	Connects phytotoxicity with bioavailability and rhizosphere chemistry	[21]
MP aging and weathering	Explained how aging modifies MP surface reactivity	Often treated aging separately from plant uptake	Integrates aging, eco-corona formation, and rhizosphere desorption	[22]
MP–PTE co-contamination in soils	Reviewed co-occurrence and environmental risks	Frameworks often remain descriptive	Develops a conceptual framework linking MP properties, soil conditions, and plant endpoints	[23]

Accordingly, the objectives of this review are: (1) to systematically evaluate how MP properties (polymer type, particle size, shape, aging state, and concentration) influence PTE adsorption, desorption, mobility, and reversibility in plant soil systems; (2) to synthesize the soil biogeochemical mechanisms—including pH shifts, dissolved organic matter (OM), redox-potential changes, mineral competition, aggregation, and microbial community restructuring—through which MPs alter PTE bioavailability; (3) to assess the consequences of MP–PTE co-exposure for plant uptake and phytotoxicity, including seed germination, root elongation, biomass production, oxidative stress responses, and metal accumulation in edible tissues; and (4) to test and refine a thematic structure that links MP properties, soil conditions, rhizosphere processes, and plant traits to explain when MPs are likely to act as sinks, vectors, or indirect modifiers of PTE exposure. This structure was initially derived from identified gaps in existing reviews (Table 1) and is evaluated here through systematic literature synthesis rather than being presented as an established theoretical framework. By addressing these objectives, we aim to provide a mechanism-based conceptual understanding that can inform risk assessment frameworks and management strategies for MP–PTE co-contamination in agricultural systems.

## 2. Review Scope and Methodological Approach

This review focuses on terrestrial plant–soil systems where MPs co-occur with PTEs. The scope includes frequently studied cationic metals such as Cd, Pb, Cu, Zn, Ni, and Cr, as well as metalloid and redox-sensitive elements such as As and Hg. These elements differ substantially in speciation, sorption behavior, mobility, and plant uptake pathway; therefore, they are not treated as a single homogeneous pollutant class. Organic contaminants, nutrients, and additives are discussed only as co-contaminants or modifiers of MP–PTE interaction, not as PTEs themselves. The review therefore uses these eight elements as analytically representative cases for cationic metals and metalloid oxyanions that differ in speciation, mobility, and root uptake behavior [15,17,24].

To improve transparency, the review scope was refined around evidence retrieved from Web of Science, Scopus, and Google Scholar. The literature search covered publications from January 2000 to February 2026. This timeframe was selected to capture early foundational work on MP contaminant interactions while emphasizing the rapid expansion of plant–soil MP–PTE research during the last decade. Search terms included combinations of terms such as “microplastics”, “soil”, “plant”, “rhizosphere”, “adsorption”, “desorption”, “bioavailability”, “phytotoxicity”, and the names of individual PTEs. Priority was given to peer-reviewed studies on plant–soil systems, including batch sorption experiments only when they directly informed mechanistic interpretation of soil outcomes. Aquatic-only studies, purely polymer-engineering papers, and papers without clear relevance to plant exposure were used sparingly or excluded from the core discussion. The core evidence synthesis was restricted to peer-reviewed journal articles. Non-peer-reviewed and gray literature, including reports, theses, preprints, and non-indexed documents, were not used to support mechanistic conclusions. Such sources were considered only when peer-reviewed evidence was unavailable for contextual information, such as terminology, policy background, or general description of MP sources and pathways.

Because field-scale and environmentally realistic plant–soil studies remain comparatively limited, this review includes mechanistic sorption studies, soil incubations, column transport studies, pot and greenhouse experiments, and selected field observations. However, evidence from simplified systems was interpreted cautiously and was not treated as equivalent to field-scale evidence. Throughout the review, solution chemistry is discussed only where it helps explain subsequent soil behavior, such as pH-dependent sorption, ionic-strength effects, competitive desorption, or the influence of dissolved organic matter on transfer into the rhizosphere. This limitation is also acknowledged in the section on future research.

A pragmatic operational meaning of bioavailability is used to maintain conceptual consistency. Bioavailability is treated as the fraction of the contaminant pool that can interact with biological uptake systems over a relevant time window, which includes pore-water species and labile solid-phase pools able to resupply pore water during uptake. This is why diffusive gradients in thin film (DGT), pore-water analysis, calcium chloride (CaCl_2_) extraction, diethylenetriaminepentaacetic acid (DTPA) extraction, ethylenediaminetetraacetic acid (EDTA) extraction, and sequential fractionation may yield different but complementary views of the same system.

## 3. Sources, Types, and Properties of Microplastics in Soils

### 3.1. Sources and Input Pathways

**Figure 1:**
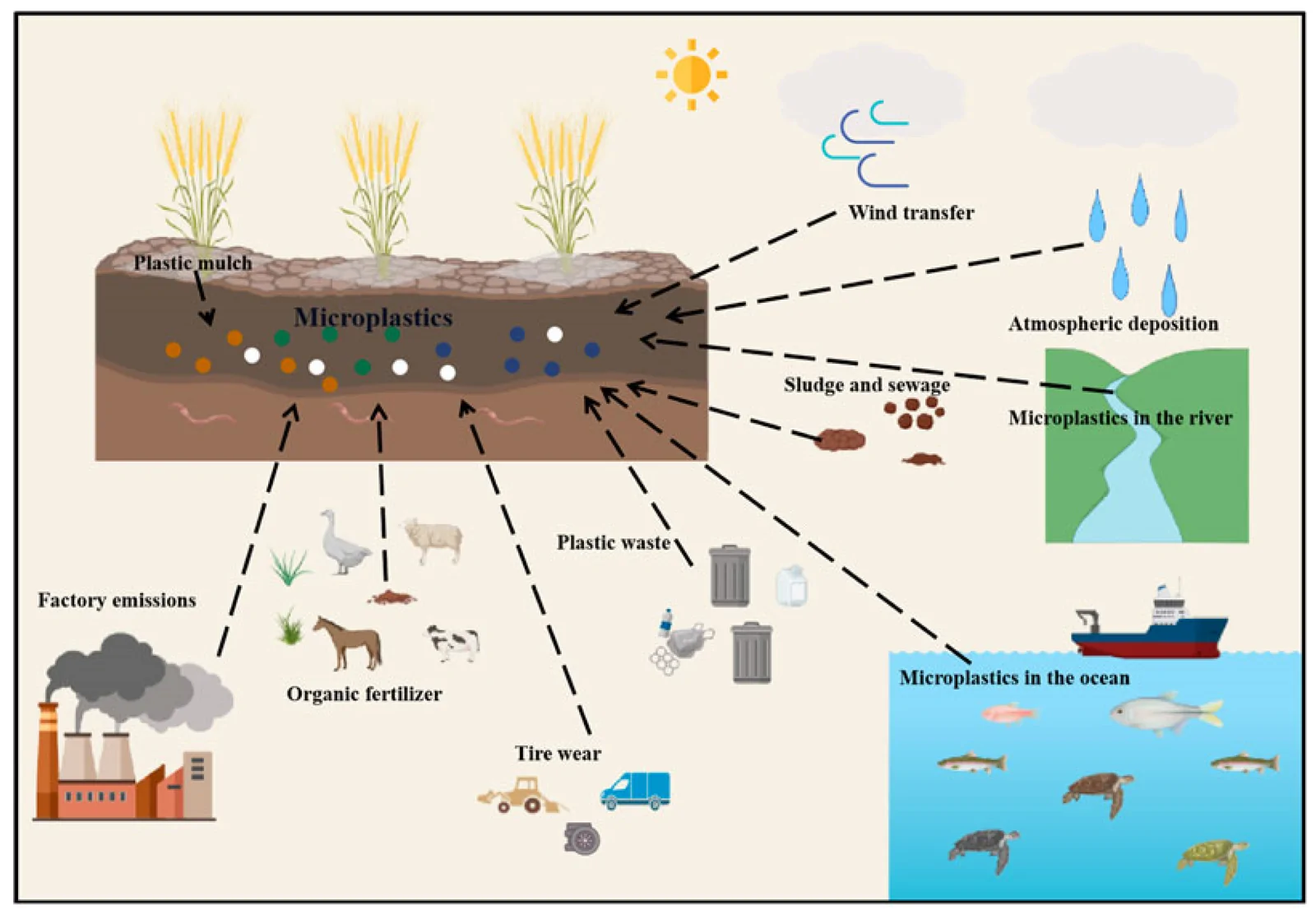
Major sources and pathways supplying microplastics to soils, emphasizing the dominant terrestrial entry routes discussed in this section, including agricultural mulch films, plastic waste, sludge and sewage inputs, organic fertilizers, atmospheric deposition, tire-wear particles, and transfers between aquatic and terrestrial compartments (adapted from [29], under the terms of the Creative Commons Attribution License (CC BY)).

Microplastics enter soils through multiple input streams whose relative importance depends on land use, waste handling, and hydrology (Figure 1). In agricultural settings, mulch films, greenhouse covers, wastewater irrigation, biosolids, organic amendments, atmospheric fallout, and roadside inputs repeatedly appear as dominant pathways. Field reports also show that fibers and fragments often predominate, whereas films are particularly common in intensively mulched vegetable systems [2,25,26].

Roadside and industrial sources represent additional but spatially variable sources of MP in soils. Roadside environments receive tire-wear particles, road-making fragments, synthetic fibers, and traffic-derived dust, which may co-occur with metals such as Zn, Cu, Pb, and Cd derived from tire additives, brake wear, fuel residues, and deposited atmospheric particles. Industrial areas can contribute pellets, fragments, fibers, pigments, stabilizers, and polymer processing residues, depending on local manufacturing, waste handling, and runoff pathways. However, the relative contribution of these sources varies strongly with land use, distance from roads or industrial facilities, stormwater transport, atmospheric deposition, and soil management practices. Therefore, roadside and industrial inputs are treated here as context-dependent co-contamination pathways rather than universal dominant sources of soil contamination. Tire-wear particles are especially relevant at the urban–agricultural interface because they contribute both plastic-like particles and Zn-rich additives, increasing the likelihood of co-delivery of MPs and PTEs to drainage systems and adjacent soils [24].

### 3.2. Morphological Characteristics and Their Environmental Significance

Soil MPs are commonly reported as fibers, fragments, films, foams, and pellets; however, their relative abundance depends strongly on sampling location, extraction method, particle-size cutoff, and identification techniques [25]. The morphological diversity of these particles is not merely an inventory detail—it directly determines their environmental behavior.

Fibers are frequently associated with wastewater, biosolids, atmospheric deposition, and textile sources. Their high aspect ratio promotes entanglement, bridging, and retention in soil pore spaces [1,27]. Films are typical of plastic mulching and greenhouse residues, providing broad contact surfaces but fragmenting into smaller particles during weathering [28]. Fragments often originate from the breakdown of larger plastic debris and exhibit irregular shapes with variable surface roughness [28]. Foams and pellets are more source-specific, usually linked to packaging, industrial leakage, or plastic processing losses. Foams contain porous structures that increase the apparent surface area, whereas pellets are often less reactive when pristine but become more relevant after aging, cracking, or biofilm formation [1,28]. Because these forms differ in aspect ratio, flexibility, roughness, density, and tendency to aggregate, particle morphology can influence pore transport, contact with mineral surfaces, biofilm formation, and the accessibility of sorption sites. These descriptors are not trivial inventory details: they determine specific surface area, mobility through pores, attachment to aggregates, and the likelihood of eco-corona formation. This is why source description must precede mechanistic interpretation in the sections that follow.

### 3.3. Polymer Type and Degradability

Compared with conventional polymers such as polyethylene, polypropylene, and polystyrene, biodegradable MPs such as polylactic acid, polybutylene adipate terephthalate, and starch-based blends may undergo faster fragmentation, surface hydrolysis, and microbial degradation in soil. However, this enhanced degradability does not necessarily reduce their PTE interaction potential; rather, the increased surface functionalization during biodegradation can create additional metal-binding sites, making polymer type a critical variable in MP–PTE co-contamination dynamics.

### 3.4. Size, Shape, and Mechanistic Implications for MP–PTE Interactions

Across soil surveys, fibers and fragments are most frequently reported, while film residues become prominent in mulched agroecosystems and finer particles become more influential for mobility and surface reactivity. Smaller particles generally provide a greater surface area and more opportunities for contact with root-associated biofilms, but they are also more strongly affected by aggregation and coating formation in real soils [30].

Polymer composition remains important, but it should not be treated in isolation. Polyethylene and polypropylene are often weak metal sorbents when pristine, whereas more polar polymers or weathered surfaces tend to exhibit stronger complexation behavior. In soil, the effective sorbent is frequently a composite interface made of polymer, mineral fines, dissolved organic matter, and microbial products rather than bare plastic alone [10,22].

Table 2 summarizes these sources in a way that directly supports later sections: the input pathway influences typical particle form, the associated additives or coatings, and the likely route by which metals become linked to MPs. In that sense, source history is an exposure determinant, not merely a descriptive background variable.

## 4. Priority PTEs in Plant–Soil MP Interaction Studies

Recent plant–soil studies do not cover all PTEs equally. Cadmium dominates the literature because it is highly bioavailable in many soils; strongly relevant to crop safety; and sensitive to pH, dissolved organic carbon, and competitive uptake pathways [37]. Arsenic also receives major attention in rice systems because its oxyanion chemistry and transporter overlap with phosphate generate distinct behavior relative to cationic metals [38]. Pb, Cu, and Zn appear regularly, while Ni, Cr, and Hg are less frequently studied but remain mechanistically important because they widen the range of valence states, nutrient interactions, and redox controls represented in the evidence base [17,18].

### 4.1. Cadmium: MP-Enhanced Mobility and Root-Zone Competition

Cadmium is repeatedly used as the model case because it is readily mobilized in weakly buffered soils and because plant uptake responds strongly to changes in pH, DOC, exchange sites, and root-zone transport competition with Zn and Ca [18,39,40]. MPs alter Cd dynamics specifically by: (i) competing for exchange sites on clay minerals and organic matter, displacing Cd^2+^ into the dissolved phase; (ii) acting as mobile vectors when Cd-sorbed MPs migrate through soil pores to the rhizosphere; and (iii) modifying DOC quality through release of polymer-derived organic compounds that form soluble Cd complexes. Studies in lettuce, wheat, and maize demonstrate that polyethylene and polystyrene MPs increase dissolved Cd concentrations by 15–40% in the rhizosphere, enhancing root uptake relative to MP-free controls [18,39,40]. Additionally, MP-induced aggregate disruption exposes previously occluded Cd-bearing surfaces, increasing the resupply-capable pool during root demand.

### 4.2. Lead: MP-Mediated Particle-Bound Transport and Rhizosphere Acidification

Lead behaves differently because much of it is retained on oxides, phosphates, and carbonates, making its availability more dependent on localized rhizosphere acidification, surface adhesion, and particle-bound transport [14]. MPs alter Pb dynamics by: (i) providing alternative sorption surfaces (carboxyl, hydroxyl groups on aged MPs) that compete with soil oxides for Pb^2+^, potentially mobilizing Pb from stable mineral phases; (ii) facilitating particle-bound transport of Pb-sorbed MPs through preferential flow paths to the root surface; and (iii) altering rhizosphere pH through release of acidic degradation products or microbial fermentation byproducts, which solubilizes Pb from carbonate and phosphate precipitates. Cu and Zn occupy an intermediate position: both are essential nutrients but can become toxic when MP-induced changes in organic-matter interactions or exchange equilibria alter the labile pool [14].

### 4.3. Nickel, Chromium, Arsenic, and Mercury: Valence, Oxyanion, and Microbial Mediation

Ni, Cr, As, and Hg are less numerous in the dataset but important for comparative interpretation. Chromium and arsenic demonstrate how valence and oxyanion chemistry complicate simple cation-adsorption models, while mercury highlights the role of microbial mediation and organic binding [15,17]. MPs alter these metalloid dynamics by: (i) modifying redox conditions in microaggregates through oxygen consumption during polymer degradation, which shifts Cr(VI)/Cr(III) and As(V)/As(III) equilibria; (ii) competing with phosphate for iron oxide sorption sites, increasing AsO_4_^3−^ mobility; and (iii) altering microbial community structure in the MP-associated plastisphere, which affects Hg methylation/demethylation rates. For that reason, the review does not treat all PTEs as equivalent; it uses the recurring evidence base to compare mechanisms across chemically distinct contaminants [15,17].

### 4.4. General Mechanism: Why Total Soil Concentration Fails to Predict Plant Exposure

Across the reviewed studies summarized in Table 3, plant exposure depends more directly on PTE lability, speciation, and resupply to pore water than on total soil concentration alone. This conclusion arises from evidence showing that MP effects are mediated through: (i) sorption/desorption shifts—MPs compete with soil minerals for PTE binding, increasing dissolved concentrations when MP sorption capacity exceeds soil retention; (ii) rhizosphere modification—MPs alter root exudation patterns, microbial community composition, and localized pH, which changes PTE solubility at the root–soil interface; (iii) aggregate redistribution—MP incorporation disrupts soil structure, exposing occluded PTE pools and altering diffusion pathways to roots; and (iv) particle-bound transport—MPs act as vectors delivering sorbed PTEs directly to the rhizosphere, bypassing bulk soil equilibrium [13,19]. What matters for plant uptake is the portion that remains dissolved or can be resupplied to pore water during root demand, which is exactly the domain where MPs, mineral surfaces, and rhizosphere ligands interact most strongly.

### 4.5. Metalloid-Specific MP Interactions

Arsenic and mercury are typically classified as metalloids and present unique behaviors. MPs alter As dynamics because As exists primarily as oxyanions (AsO_4_^3−^ and AsO_3_^3−^), so its interactions involve competitive sorption with phosphate on iron oxides rather than simple cation exchange. MPs can displace As from oxide surfaces through competitive sorption of polymer-derived organic anions or enhance As mobility by increasing pore-water DOC that forms soluble As-organic complexes. MPs alter Hg dynamics because Hg exists in multiple forms. including methylated species (MeHg) in biologically active zones; MP-induced changes in microbial community structure within the plastisphere can enhance or suppress mercury methylation rates, while MP-associated biofilms provide organic binding sites that sequester Hg^2+^ or facilitate its reduction to volatile Hg^0^ [18].

### 4.6. Plant Exposure Pathways and MP-Mediated Fraction Shifts

For plant exposure, pathways include uptake from pore water into roots, adsorption on root surfaces, and translocation to shoots and edible tissues. The fraction that becomes plant-accessible is shaped by root exudation, microbial activity, and rhizosphere pH, which can locally override bulk soil conditions. MP presence shifts PTEs among geochemical fractions—soluble, exchangeable, carbonate-bound, reducible, oxidizable, and residual—through: (i) competitive sorption on MP surfaces depleting exchangeable and carbonate pools, (ii) redox modification in MP-microaggregate microsites mobilizing reducible/oxidizable fractions, and (iii) physical disruption of aggregates releasing residual-phase PTEs to more labile pools. These MP-driven fraction shifts change both short-term (days) and longer-term (weeks) plant exposure dynamics beyond what total soil concentration would predict [2].

### 4.7. Basis for Selecting Priority PTEs

The PTEs selected for this review were identified through iterative screening of the MP–soil–plant literature to capture the most informative and representative cases for understanding MP-mediated processes. Rather than applying arbitrary inclusion thresholds, we employed three pragmatic principles grounded in the actual structure of the available evidence:

Principle 1: Coverage of dominant evidence bases. Cd and As were prioritized because they constitute the most extensively documented cases in MP–soil–plant co-exposure studies, particularly for Cd in leafy vegetables and cereals and for As in flooded rice systems where MP–iron plaque interactions are well characterized. These elements provide the empirical foundation for assessing how MPs alter established soil–plant transfer pathways.

Principle 2: Representation of contrasting chemical behaviors. Pb, Cu, and Zn were included to encompass the spectrum of cationic metal behavior: Pb represents strongly sorbed, low-mobility metals dependent on rhizosphere acidification; Cu represents micronutrients with high organic-matter affinity and redox sensitivity; and Zn represents more labile, exchangeable metals that compete with Cd for uptake transporters. Together, these three capture the range of binding strengths, nutrient essentiality, and toxicity thresholds observed for divalent cations in MP-amended soils.

Principle 3: Inclusion of mechanistically distinct outlier cases. Ni, Cr, and Hg were retained despite their lower representation in the MP–plant–soil literature because they introduce critical chemical behaviors not represented by the higher-frequency elements: Ni demonstrates pH-dependent exchange dynamics on clay minerals, Cr introduces redox-sensitive valence-state transitions (Cr(III)/Cr(VI)) that challenge simple cation-exchange models, and Hg requires consideration of microbial methylation and organic complexation pathways that operate independently of mineral surface sorption. Their inclusion ensures the review addresses MP effects on redox, microbial, and organo-metallic processes, not just conventional cation adsorption. Thus, these eight PTEs constitute a purposive sample designed to maximize mechanistic coverage rather than a comprehensive inventory. They are discussed in Section 8 in groupings that reflect their chemical similarities: labile cationic metals (Cd and Zn), strongly retained cationic metals (Pb and Cu), oxyanion-forming elements (As and Cr), and microbially mediated elements (Hg and Cr).

To make the “strong” versus “limited” evidence labels used above more transparent, an approximate indication of relative literature density is given here rather than a formal systematic count: a Web of Science/Scopus title–abstract search combining “microplastic*” with “soil” and each PTE name (2015–2026) returns on the order of several dozen plant–soil-relevant records for Cd and, for As, a smaller but still double-digit set for Pb, Cu, and Zn and only a handful of directly relevant plant–soil records for Ni, Cr, and Hg, most of which address these elements as secondary or comparative cases rather than as the primary study focus. These figures are approximate and search-strategy-dependent, but they are consistent with the qualitative “strong/moderate/limited” evidence-status labels assigned in Table 3, and they clarify that the labels reflect relative publication density rather than an assumption about the true environmental importance of each element.

## 5. Adsorption, Desorption, and Reversibility of PTEs on MPs

Microplastics can function as sorbents for PTEs, but the literature shows wide variation in apparent capacity, reversibility, and mechanism. In pristine systems, adsorption of Cd to PE can be relatively low and often fits Langmuir-type behavior; one commonly cited aqueous study estimated a maximum Cd adsorption capacity of 30.5 μg g^−1^ for PE particles, while desorption remained readily reversible [43]. In contrast, aged, oxidized, mineral-coated, or biofilm-bearing surfaces typically show stronger and more heterogeneous binding, which is better captured by Freundlich-like behavior or by multi-stage kinetic models [16,42].

This variability explains why adsorption should be reviewed as an evidence pattern rather than as a fixed property of polymer identity. In plant–soil systems, MPs compete with clay minerals, Fe/Mn oxides, and organic matter for the same PTE pool, so reported adsorption parameters often describe system redistribution rather than sorption to a chemically isolated plastic surface [15].

**Figure 2:**
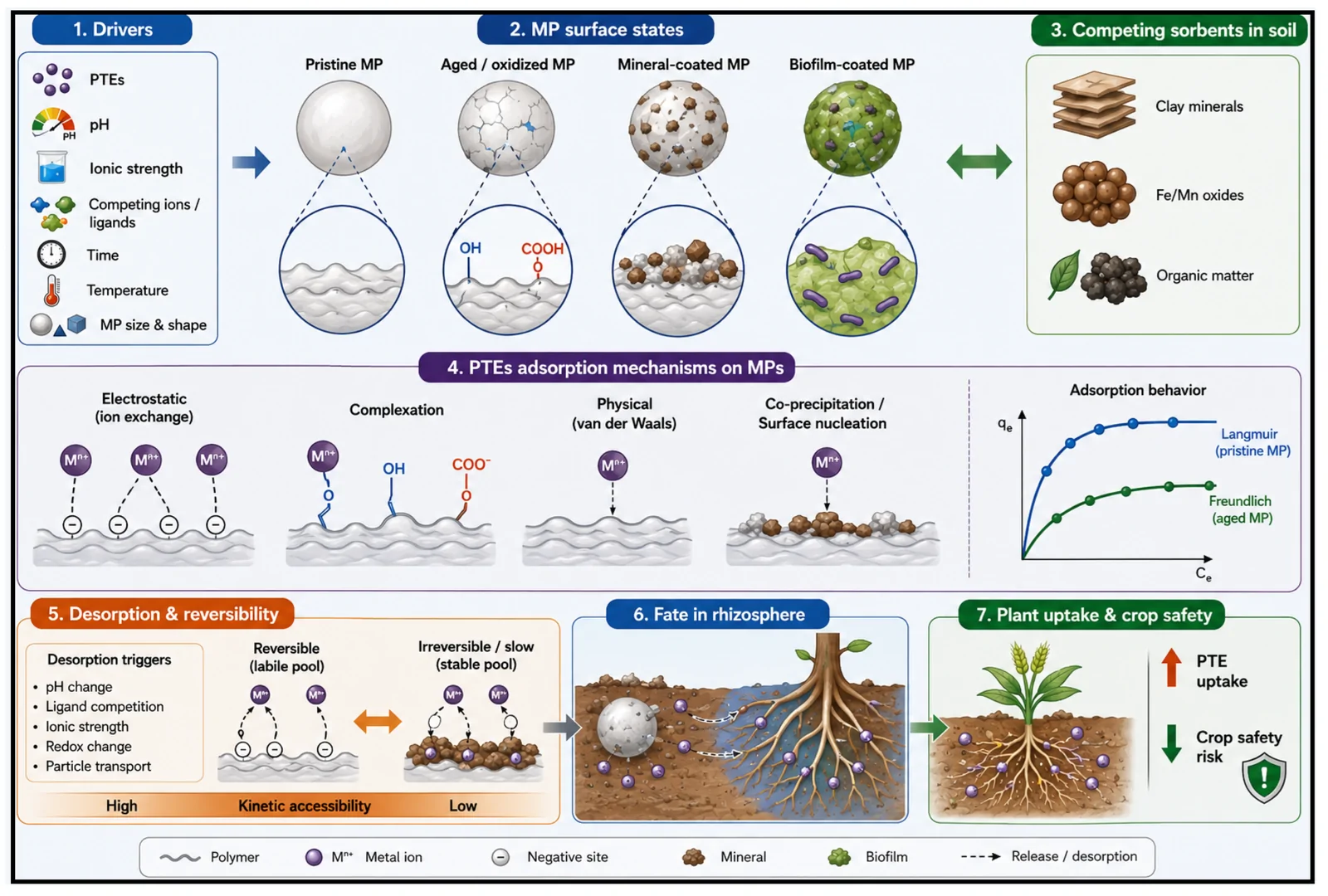
Conceptual framework of PTE adsorption and desorption on microplastics (MPs), highlighting surface transformation, soil interactions, reversibility, and plant exposure. Created by the authors based on concepts reported in [4,13,15,16,19,42,43,44,45,46].

Physical adsorption contributes through van der Waals forces and hydrophobic partitioning of organo-metal complexes, yet for cationic metals, the dominant contributions in many systems arise from electrostatic attraction, ion exchange, and complexation with functional groups such as carbonyl, hydroxyl, and carboxyl moieties generated by oxidation or biofilm conditioning. In soils, co-precipitation and surface nucleation can occur when metals interact with mineral coatings on MPs, blurring the distinction between adsorption to plastic and adsorption to the attached mineral phase. As summarized in Figure 2, these processes are controlled jointly by solution chemistry, MP surface condition, and competition with natural soil sorbents, while subsequent desorption determines whether the retained PTE pool remains kinetically accessible for transport toward the rhizosphere and plant uptake.

Studies comparing pristine and environmentally conditioned MPs show that surface aging, oxidation, wastewater conditioning, and biofilm or eco-corona formation can increase sorption heterogeneity and alter desorption behavior (Table 4). For example, UV-aged MPs generally show greater surface roughness and oxygen-containing functional groups than pristine MPs, wastewater conditioned MPs can exhibit enhanced metal adsorption potential, and biofilm-coated microfibers can provide additional extracellular polymeric binding domains for metal retention. These comparisons indicate that the strongest contrast is often not simply among polymer classes but between pristine particles and environmentally transformed particles [4,45]. Particle morphology also matters because fibers, foams, and cracked films can create microdomains that retain colloids and extracellular polymers. Those microdomains may increase apparent sorption and slow desorption, but they can also preserve kinetically accessible PTE pools that later become available in the rhizosphere.

The key review point is therefore that sorption strength alone does not predict bioavailability. Stronger adsorption can coincide with higher plant exposure when the adsorbed pool remains labile under root-driven pH change, ligand competition, or particle transport into root-active zones [13,19,46].

### 5.1. Reported Isotherm and Kinetic Patterns

Across the reviewed MP–PTE adsorption studies, Langmuir and Freundlich models are the most frequently applied equilibrium isotherms, but they serve different interpretive purposes. Langmuir-type fits are generally used to describe finite-site adsorption and apparent saturation behavior, especially in simplified aqueous or single sorbent systems. In practice, Freundlich fits become more common once particles are aged or coated because the surface is no longer energetically uniform. Kinetic studies commonly apply pseudo-first-order, pseudo-second-order, and intraparticle diffusion models. However, these models should be interpreted cautiously: pseudo-second-order fitting is often described as evidence of chemisorption, but this inference is not definitive unless supported by surface spectroscopy or chemical speciation evidence. In soil systems, fitted isotherm and kinetic parameters may reflect redistribution among MPs, mineral surfaces, organic matter, and pore water rather than adsorption to plastic alone. Langmuir fits remain useful in simplified single-phase systems, especially for pristine polymers and restricted concentration ranges [47,48].

Kinetic interpretation also changes with environmental context. Batch systems may suggest rapid initial adsorption followed by a slower stage controlled by diffusion into surface irregularities or coatings, whereas soil systems add competition from mineral surfaces and limits on pore-water renewal. As a result, kinetic parameters are most informative when coupled with lability metrics such as DGT or with plant uptake data rather than interpreted in isolation.

Taken together, the current literature supports a simple pattern: adsorption models are useful descriptors, but their parameters are conditional on particle aging, competing sorbents, and chemical definition of the accessible pool. The review therefore uses isotherm and kinetic data to frame later discussion of soil factors rather than to treat them as self-sufficient evidence.

**Table 4:** Common adsorption models and the mechanistic interpretations used in MP–PTEs studies.

Model	Typical Equation Form (Conceptual)	Mechanistic Implication	Typical Interpretation in MP–PTE Studies	Main Limitation in Soil System	Real-World Applicability (Aqueous, Soil Incubation, and Soil–Plant Systems)	Ref.
Langmuir Equilibrium isotherm	q approaches qmax as C increases	Finite sites; saturation behavior	Often used for pristine or simplified MP systems where apparent maximum capacity is estimated	Soil nutrients, organic matter, and coating can mimic saturation behavior	Aqueous: well-suited (single-solute, controlled conditions match model assumptions). Soil incubation: moderate (competing sorbents/ions can violate single-site assumption but often used as first approximation). Soil-plant: limited (continuous root uptake violates the equilibrium assumption; rarely directly validated in planted systems).	[49,50]
Freundlich Equilibrium isotherm	q scales with C^n	Heterogeneous adsorption across non-uniform surfaces	Often used for aged, oxidized, biofilm-coated, or eco-corona-bearing MPs	Parameters are empirical and strongly context-dependent	Aqueous: commonly used, even for simple systems. Soil incubation: well-suited given natural surface heterogeneity. Soil–plant: applicable but confounded by root-driven changes in local concentration gradients.	[50]
Pseudo-first-order kinetics	Rate proportional to remaining capacity	Often diffusion or weak binding control	Associated with weak surface adsorption or diffusion-limited uptake	Agitation, pore-water renewal, and competing sorbants affect fitted rates	Aqueous: good fit for short-timescale, diffusion-limited early-stage kinetics. Soil incubation: applicable to the initial uptake phase but often deviates at longer times due to soil heterogeneity. Soil–plant: rarely tested directly; root exudates and microbial activity can alter the apparent rate	[47]
Pseudo-second-order kinetics	Rate proportional to square of remaining capacity	Often interpreted as chemisorption control	Frequently interpreted as chemisorption	Chemisorption cannot be confirmed from model fit alone	Aqueous: frequently the best-fitting model in batch studies. Soil incubation: commonly reported, but the chemisorption interpretation cannot be verified in a matrix of competing sorbents. Soil–plant: essentially unvalidated; continuous plant uptake violates the closed-system kinetic assumption	[34]
Intraparticle diffusion	q scales with t^0.5 segments	Diffusion through pores/coatings	Useful for aged or porous particles with surface irregularities	Soil aggregation and biofilm growth complicate diffusion interpretation	Aqueous: useful diagnostic for porous-particle uptake. Soil incubation: complicated by aggregation/biofilm growth but still used diagnostically. Soil-plant: least validated context; root-zone gradients and microbial colonization introduce additional diffusion barriers not captured by the model.	[51]

### 5.2. Factors Controlling Sorption, Desorption, and Reversibility

Surface functionalization is a major determinant of metal adsorption because oxidation, UV aging, abrasion, and biofilm conditioning introduce carbonyl, hydroxyl, and carboxyl groups and increase roughness. These changes enlarge the number of coordination sites and often make adsorption more sensitive to pH and ionic competition [4].

Additives and leachates can amplify or suppress binding depending on whether they expose reactive heteroatoms, release complexing ligands, or block existing sites. The practical implication is that the term “polyethylene” or “polystyrene” is often chemically incomplete unless the particle state, additive history, and environmental conditioning are also reported.

The interaction between MP-derived organic compounds, soil organic matter, and rhizosphere processes is especially important because these components can influence DOM composition, metal complexation, and root-zone desorption behavior. In PE–MP and Cd co-contaminated soybean soil, integrated metabolomic and rhizosphere microbial evidence further indicates that PE–MPs can alter Cd behavior through plant–metabolite–microbe interactions, thereby affecting Cd accumulation in roots [52].

### 5.3. Mobility and the Sink-Vector Transition

**Figure 3:**
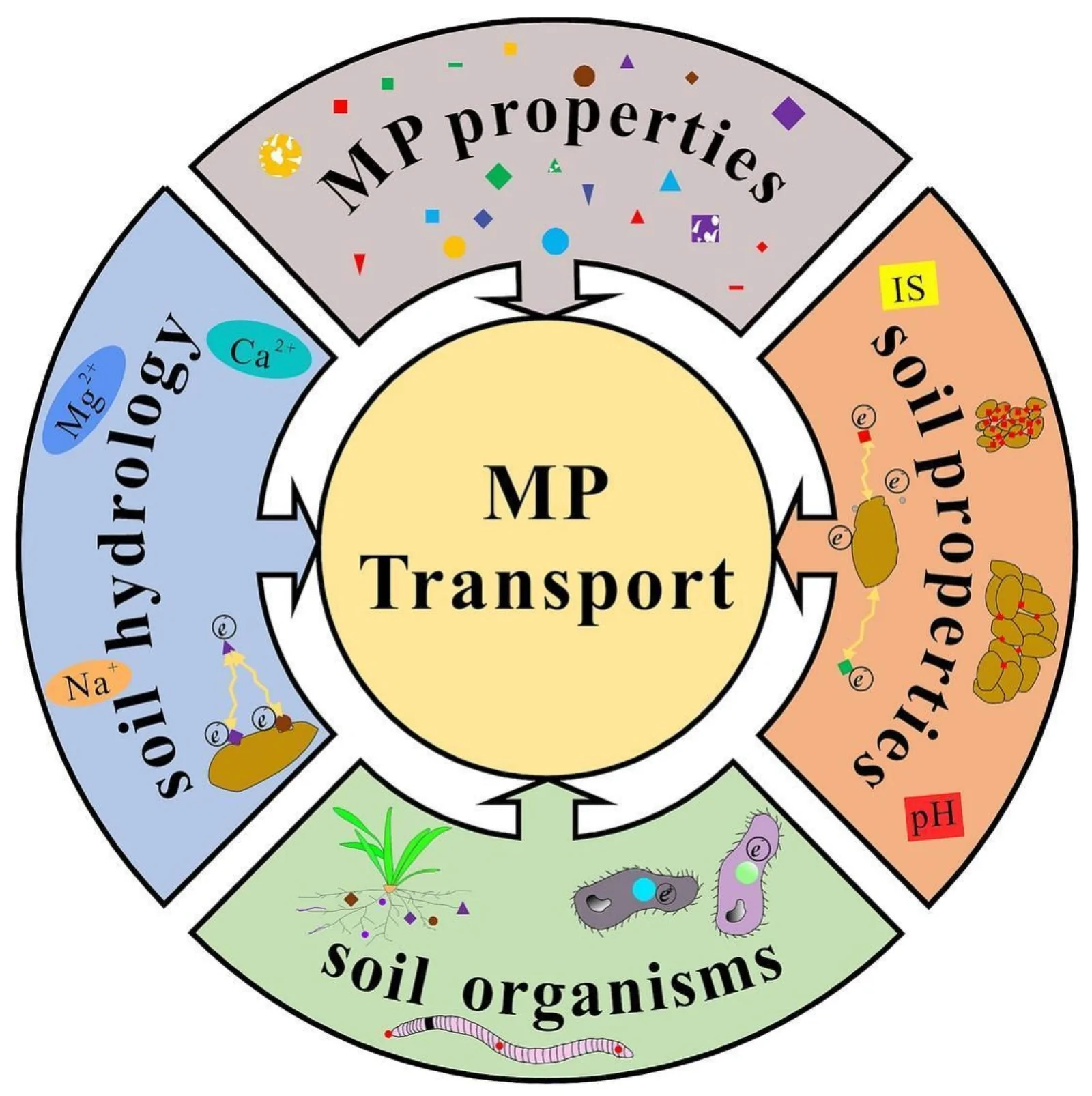
Main controls on MP transport in soils, showing how particle properties, soil physicochemical conditions, hydrology, and biological activity jointly determine whether PTE-bearing MPs are immobilized within aggregates or redistributed through pore networks (adapted from [55] under the terms of the Creative Commons CC-BY license).

Microplastics influence PTE mobility when adsorbed species remain associated with particles that can move through pore networks or along preferential flow paths. Evidence from soil and column studies indicates that this mobility depends on particle size, attachment to mineral surfaces, wetting history, and the formation of heteroaggregates rather than on polymer type alone [34,53,54] (Figure 3).

Aggregation is the pivot process. Once MPs bind to clays, oxides, or biofilms, they may become immobilized and behave as sinks; if they remain colloidally stable, they can redistribute sorbed or exchangeable PTEs across depths and toward root-active zones. This is why mobility should be read as an extension of sorption, not as a separate phenomenon.

### 5.4. Element-Specific Desorption Behavior

#### 5.4.1. Cadmium Desorption

Cd exhibits high desorption reversibility from MPs due to its weak binding affinity. PVC microplastics reduced Cd(II) adsorption on soil and enhanced desorption through competitive effects, with soil-dependent mechanisms controlling the magnitude of release. In batch experiments, PE microplastics decreased soil adsorption capacity for Cd while increasing Cd desorption, thereby increasing soil Cd mobility [56]. Zhang et al. further showed that Cd desorption from MPs was strongly influenced by particle dose, size, and solution pH, with higher desorption rates under acidic conditions due to proton competition for binding sites [57]. These findings indicate that Cd sorbed to MPs remains highly labile in soil environments, particularly in acidic or low-buffering soils where root exudation and microbial activity can further promote desorption.

#### 5.4.2. Lead Desorption

Lead demonstrates stronger adsorption but conditional desorption depending on pH and coating composition. Lin et al. indicated that Pb(II) sorption onto MPs significantly increases at pH 2.0–6.0 due to electrostatic attraction, but desorption becomes significant when pH drops below 3 or when competing cations (Ca^2+^, Mg^2+^) are present at high concentrations [58]. In soil incubation studies, Pb desorption from HDPE-MPs showed variable patterns: while some experiments reported decreased Pb sorption capacity in the presence of MPs, others found no significant impact, suggesting soil-specific controls [59].

#### 5.4.3. Copper Desorption

Copper desorption from MPs is strongly influenced by organic-matter interactions and redox conditions. Peng et al. (2024) investigated how PS microplastics affect Cu adsorption in soil and found that MPs altered Cu partitioning between soil solids and pore water, with desorption enhanced under conditions promoting DOM release [60]. LDPE, PP, and PET microplastics altered Cu extractability in four clayey–sandy soils, with slight increases in Cu availability under acidic conditions after prolonged incubation, but Cu consistently exhibited lower mobility than Cd (RAC: 1.1–3.8% vs. 6–35% for Cd) [61]. The stabilizing effect of MPs on Cu increased over time, particularly for PET, indicating that polymer type and aging jointly regulate desorption kinetics [61].

#### 5.4.4. Zinc Desorption

Zinc shows intermediate desorption behavior between Cd and Cu. Xiong et al. (2025) [61] found that microplastics enhanced ZnO nanoparticle adsorption capacity through interactive mechanisms, but desorption was significant when pH decreased or DOM concentration increased, suggesting conditional reversibility [62]. In agricultural soils, MPs reduced Zn adsorption by competing for soil binding sites, potentially increasing Zn desorption and mobility. However, Zn desorption rates were generally lower than Cd, reflecting stronger Zn retention on soil minerals and organic matter.

#### 5.4.5. Arsenic Desorption

Arsenic desorption from MPs is controlled by phosphate competition and iron oxide dynamics rather than simple cation exchange. Han et al. (2024) [62] compared As(V) and Cd(II) adsorption on biodegradable (PBSA) and non-biodegradable (LDPE) microplastics in soil [63]. PBSA increased As(V) adsorption (0.43 to 0.49 mg/g) through hydrogen bonding, while LDPE reduced As(V) adsorption (0.44 to 0.40 mg/g) due to a “dilution effect”. Desorption of As from PBSA was lower than from LDPE, indicating that biodegradable MPs may more strongly retain As through surface functional groups [63].

#### 5.4.6. Chromium and Nickel Desorption

Chromium and nickel desorption are less studied but mechanistically distinct. For Cr, desorption depends strongly on the valence state: Cr(VI) is more weakly adsorbed and more readily desorbed than Cr(III), which forms stable inner-sphere complexes with oxide surfaces [59]. MP presence can alter Cr redox microsites, potentially enhancing Cr(VI) reduction to less mobile Cr(III) or, conversely, promoting Cr(III) oxidation under certain conditions [59]. For Ni, desorption is pH-dependent and generally weak across polymer types, with HDPE-MPs showing no significant impact on Ni sorption/desorption in some studies [6]. However, Jadhav et al. showed that Ni adsorption to microplastic is strongly polymer-type-dependent, with PP showing the highest affinity, suggesting that desorption potential varies with polymer chemistry [51].

#### 5.4.7. Mercury Desorption

Mercury desorption is the least resolved due to complex organic binding and microbial methylation pathways. Chakraborty et al. [64] demonstrated that microplastics interact with Hg bioaccumulation in fish, but soil–plant studies are scarce. Hg desorption from MPs is likely mediated by biofilm-associated thiol groups, sulfide precipitation, and microbial demethylation, making simple batch desorption tests inadequate for the prediction of field behavior.

### 5.5. Soil-Based Controls on MP–PTE Mobility

Soil-based evidence further shows that MP effects on PTE mobility are controlled by interactions with native sorbents, including clay minerals, OM, and natural colloids. In fine-textured or oxide-rich soils, mineral surfaces may dominate PTE retention and reduce the relative influence of MPs. In contrast, in sandy, low-organic-matter, or weakly buffered soils, MPs can exert proportionally stronger effects on the dissolved or exchangeable PTE pool [61]. Aging and eco-corona formation add further complexity because mineral- and biofilm-coated MPs may immobilize PTEs through heteroaggregation, whereas DOM-rich coatings may stabilize MP–DOM–PTE complexes and enhance colloidal transport [61,63]. Thus, MPs should be interpreted as conditional sinks, vectors, or indirect reactivity modifiers rather than as uniform mobile carriers of PTEs [65].

## 6. Aging, Weathering, and Biofilm as Interaction Modifiers

**Figure 4:**
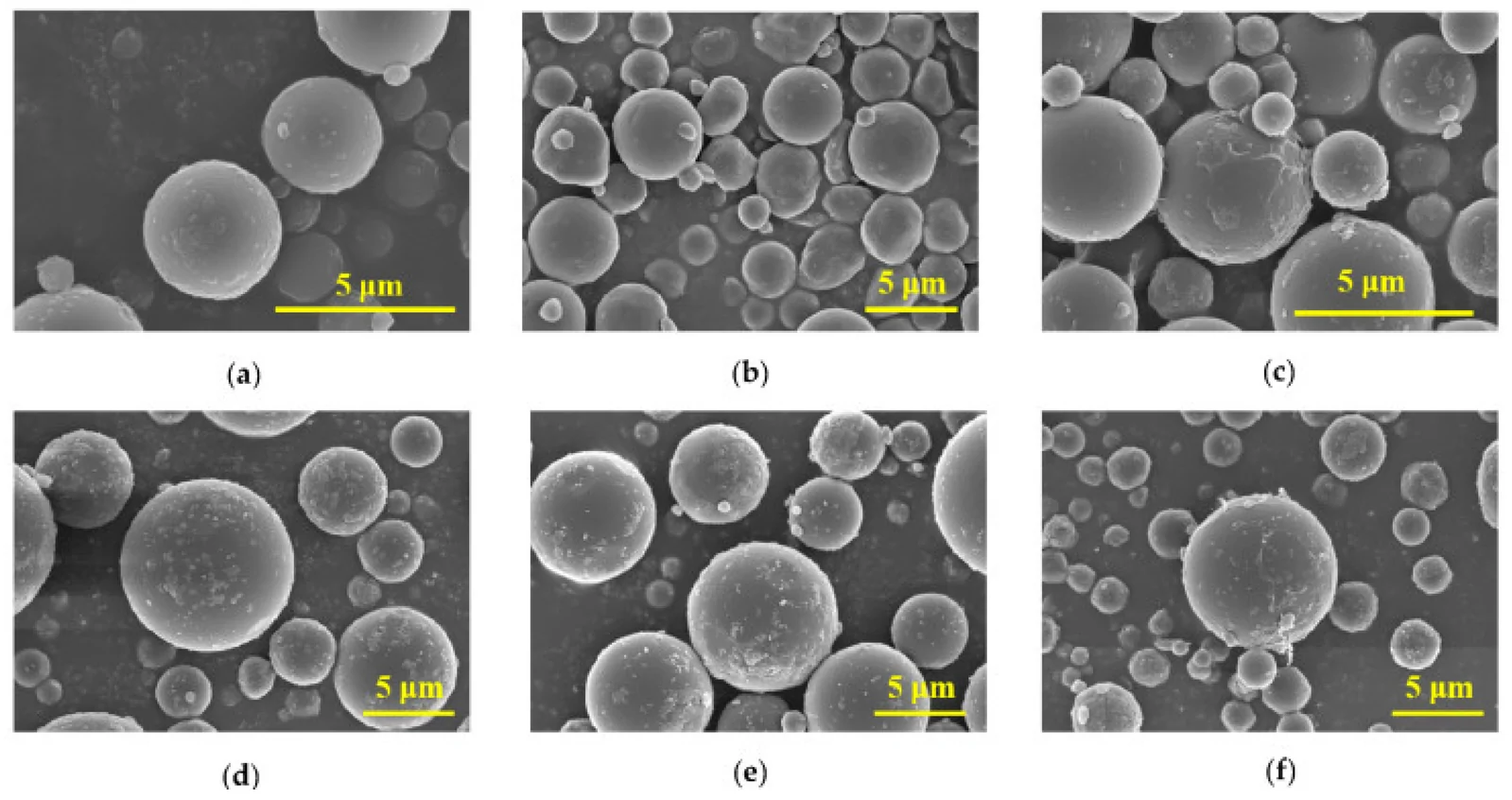
Scanning electron microscopy images of polystyrene microplastics under pristine and aged conditions: (**a**–**c**) 3 μm PS under pristine, UV-aged, and bio-aged conditions, respectively; (**d**–**f**) 10 μm PS under pristine, UV-aged, and bio-aged conditions, respectively. The images illustrate UV-driven roughening and bio-aging-associated surface modification (adapted from [48], CC BY 4.0).

Aging, weathering, and biofilm formation modify MP–PTE interactions because they convert relatively inert polymer surfaces into heterogeneous reactive interfaces (Figure 4). Pristine PE and PP generally contain few polar functional groups and therefore often show limited affinity for cationic PTEs; however, UV irradiation, thermal oxidation, mechanical abrasion, and biological aging increase surface roughness, cracking, porosity, and oxygen-containing functional groups such as carbonyl, hydroxyl, and carboxyl moieties [48,66,67,68]. These changes increase the number of potential binding domains and promote electrostatic attraction; surface complexation; cation bridging; and, in some cases, co-precipitation with mineral phases attached to the plastic surface.

### Polymer-Specific Aging Effects on Functional Groups and PTE Affinity

PE (polyethylene): UV and thermal oxidation introduce hydroxyl (-OH) and carbonyl (-C=O) groups on the linear -CH_2_– backbone. Aged PE shows increased Cd sorption capacity (up to 64.6% enhancement after 324 h UV exposure in seawater), with surface roughening and enhanced negative surface charge promoting electrostatic interactions [67].

PP (polypropylene): UV aging forms carbonyl (-C=O) and carboxyl (-COOH) groups on the methyl-branched structure. Aged PP exhibits enhanced adsorption of pharmaceuticals and metals through increased hydrophilicity and surface free energy, with the carbonyl index increasing significantly compared to virgin particles [69].

PS (polystyrene): Aging introduces hydroxyl (-OH) and carbonyl (-C=O) groups on the aromatic -CH_2_-CH(Ph)– backbone. PS shows moderate adsorption enhancement after oxidation, with aged particles developing more reactive sites for metal binding.

PVC (polyvinyl chloride): The chlorine-containing -CH_2_-CH(Cl)- structure promotes inherent surface reactivity; oxidation adds C=O and -COOH groups. PVC demonstrates high affinity for metals like Pb and Cd due to this combined halogen and oxygen functionality.

PET (polyethylene terephthalate) contains inherent carbonyl ester groups in its backbone, making it less responsive to further oxidation-induced functional-group enhancement. PET shows minimal adsorption increase after aging compared to other polymers, as its native functionality already dominates surface chemistry [67].

Empirical studies using SEM, FTIR, XPS, zeta-potential analysis, adsorption isotherms, and desorption tests consistently show that aged or oxidized MPs usually adsorb more Cd, Pb, Cu, Zn, and other PTEs than pristine particles, although the magnitude depends strongly on polymer type, aging pathway, pH, ionic strength, dissolved organic matter, and competing ions [42]. Therefore, aging should not be treated simply as physical degradation; it directly changes the chemical reactivity, reversibility, and environmental role of MPs in contaminated soils.

The effect of coating is especially important because MPs in soils are rarely present as bare polymers. Under field-relevant conditions, aged MPs rapidly acquire mineral, organic matter, and microbial coating, forming composite interfaces composed of polymer, clay minerals, Fe/Mn oxides, carbonates, humic substances, dissolved organic matter, and extracellular polymeric substances [70]. Mineral-rich coating can increase the effective density and attachment efficiency of MPs by adding mineral mass, modifying surface charge, and promoting cation bridging between MPs and soil colloids. This favors heteroaggregation with clay minerals, Fe/Mn oxides, and organic matter, thereby reducing pore-scale transport and promoting retention within soil pores or aggregates [55]. In such cases, MP-associated PTEs may become immobilized in less labile pools, especially when aggregate-level analyses, pore-water measurements, sequential extraction, or DGT-based methods show decreases in soluble or exchangeable metal fractions. However, this immobilization effect is conditional rather than universal. DOM-rich coatings may stabilize MP–DOM–PTE colloids, maintain metals in exchangeable forms, or facilitate transport towards root-active microsites, meaning that coated MPs can either reduce or enhance PTE bioavailability depending on coating composition, soil mineralogy, hydrology, and rhizosphere chemistry [53].

Biofilm formation adds another layer of complexity because microbial extracellular polymeric substances provide abundant carboxyl, phosphate, hydroxyl, amine, and sulfhydryl groups that can bind PTEs, while microbial respiration creates microscale pH and redox gradients around particles [71].

Overall, available empirical evidence indicates that aging, weathering, mineral attachment, eco-corona formation, and biofilm development modify MP–PTE interactions through four linked mechanisms: increased reactive surface area, the introduction of new functional groups, promotion of mineral–organic heteroaggregation, and alteration of desorption under rhizosphere conditions. These processes determine whether MPs behave mainly as PTE sinks, mobile vectors, or indirect modifiers of PTE bioavailability.

## 7. Soil Factors Controlling Adsorption, Mobility, and Exposure

Soil physicochemical conditions control not only PTE speciation but also whether adsorption to MPs is strong, weak, or readily reversible. The following subsections address how each major soil factor influences MP–PTE interactions across all priority elements (Cd, Pb, Cu, Zn, As, Cr, Ni, and Hg), not only the most frequently studied cases.

Soil pH: Soil pH governs protonation of reactive surface groups and the solution chemistry of many cationic metals; consequently, lower pH often increases dissolved PTE fractions and can favor desorption or exchange near roots, whereas neutral to alkaline conditions may shift metals toward oxide-bound or precipitated forms that compete with MP sorption [15,71].

Cadmium provides a useful example of how pH controls PTE speciation, adsorption, and mobility. Under acidic conditions, Cd occurs mainly as free Cd^2+^ and weakly complexed soluble forms. At the same time, excess H^+^ competes with Cd^+2^ for negatively charged exchange sites on the clay minerals, organic matter, Fe/Mn oxides, and oxidized MP surfaces. Protonation of carboxyl, hydroxyl, and oxide surface groups reduces negative surface charge, weakens Cd adsorption, and increases the exchangeable and pore-water Cd fractions. As a result, Cd mobility and root exposure generally increase in acidic soils [72].

As pH rises towards neutral or alkaline conditions, surface deprotonation increases the number of negatively charged binding sites on minerals, OM, and aged MP surfaces. Cd adsorption therefore increases through electrostatic attraction, cation exchange, and surface complexation. In carbonate-rich or alkaline soils, Cd may also shift toward hydrolyzed, carbonate-associated or precipitated forms, including CdOH^+^, CdCO_3_^0^, CdHCO_3_, or Cd(OH)_2_, depending on the carbonate availability and solution chemistry. These reactions generally reduce Cd mobility by transferring Cd from soluble and exchangeable pools into more strongly adsorbed or precipitated fractions. However, this immobilization can be reversed locally in the rhizosphere when root-derived protons, organic acids, dissolved organic ligands, or microbial activity remobilize Cd from MP-associated, mineral-associated, or carbonate-bound pools.

Ionic strength and electrolyte composition: Ionic strength and electrolyte composition modify double-layer thickness, cation bridging, and heteroaggregation. In practical terms, they help determine whether metal-bearing MPs remain suspended and exchangeable or become attached to mineral colloids and aggregate surfaces, thereby altering the apparent adsorption parameters reported in soil systems [73].

For Cd, Pb, Cu, and Zn, higher ionic strength (e.g., saline soils or fertilizer applications) compresses the electrical double layer, reducing electrostatic repulsion between MPs and mineral surfaces, promoting heteroaggregation, and potentially immobilizing MP-associated metals in less labile pools [9]. Conversely, low ionic strength favors MP dispersion and metal transport. As and Cr oxyanions show contrasting behavior: increased ionic strength can enhance anion adsorption through specific surface complexation while suppressing electrostatic adsorption, making net effects difficult to predict. Hg mobility is strongly influenced by chloride complexation at high ionic strength (formation of HgCl_2_^0^ and HgCl_3_^−^), which increases Hg mobility and bioavailability [5].

Redox Conditions: Redox conditions add another layer of control because Fe/Mn oxides, Cr valence, and As speciation all respond to changes in oxygen availability. MPs may not dominate these reactions directly, but they can influence microsites for microbial colonization and thereby change where reduction, dissolution, or secondary sorption occurs inside aggregates [15]. Chromium is the most redox-sensitive element: Cr(VI) is mobile and toxic, while Cr(III) is less mobile and less bioavailable. MPs can create localized anoxic microzones through oxygen consumption during polymer degradation or microbial respiration in biofilms, potentially enhancing Cr(VI) reduction to Cr(III). Arsenic speciation shifts between As(V) (less mobile, adsorbed) and As(III) (more mobile, toxic) under reducing conditions; MP-associated microbial communities can mediate this transition. Mercury methylation (Hg^2+^ → MeHg^+^) is strongly redox-dependent and microbially mediated; MP biofilms may enhance or suppress methylation rates depending on microbial community composition. Cd, Pb, Cu, and Zn are less directly redox-sensitive but can be released from Fe/Mn oxides when these minerals dissolve under reducing conditions, potentially increasing bioavailability despite being in less labile pools under oxic conditions.

Moisture fluctuations and wetting–drying cycles further link adsorption to mobility. Rewetting can release colloids and dissolved PTEs from aggregates, while drying can concentrate solutes and promote closer particle–surface contact. These dynamics make exposure episodic, especially in irrigated systems or surface horizons receiving plastic residues [4,53].

Texture, mineralogy, and soil organic matter determine whether MPs are minor auxiliary sorbents or locally important reactive phases. In fine-textured or oxide-rich soils, mineral surfaces may dominate PTE retention; in sandier or lower-organic-matter systems, MPs can exert proportionally larger effects on lability and plant exposure [17,33,74].

### 7.1. Dissolved Organic Matter and Natural Colloids

Dissolved organic matter strongly influences both adsorption and release. DOM can bind PTEs in solution, compete for surface sites, or coat MPs to form new functional domains. In consequence, DOM may either reduce free-ion activity or create mobile MP–DOM–PTE associations that redistribute labile pools through soil pores [10,17].

**Figure 5:**
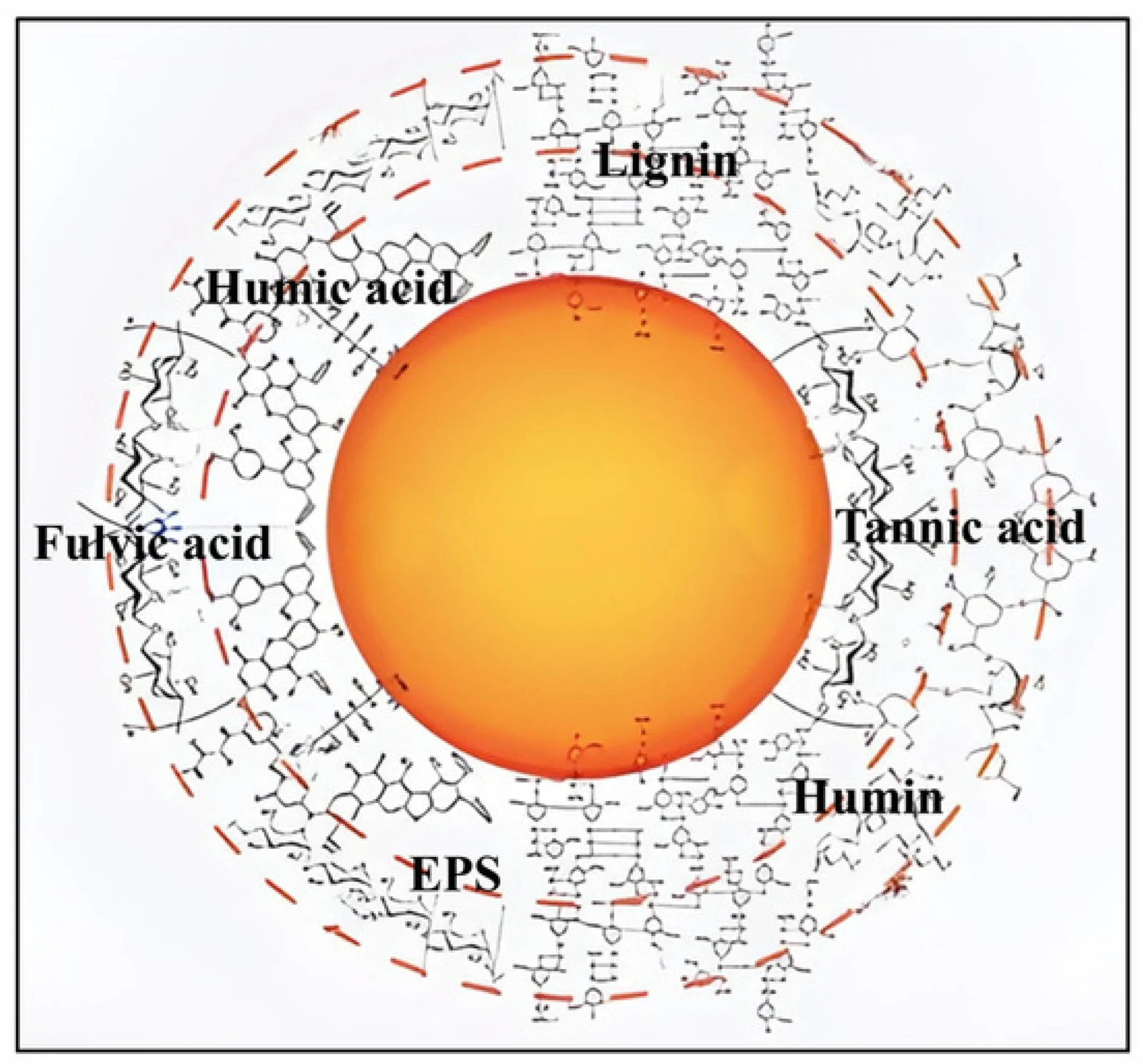
Eco-corona concept applied to MPs in soils: dissolved organic matter, extracellular polymeric substances, and mineral colloids can coat particle surfaces and create new reactive domains that change colloidal stability, heteroaggregation, and PTE binding behavior (adapted from [75], CC BY 4.0).

Ternary associations among MPs, DOM, and metals are therefore central to interpretation. Under some conditions, they increase transport by stabilizing colloids; under others, they immobilize metals by promoting attachment to clays or Fe oxides. This duality is discussed here because eco-corona formation—the coating of MP surfaces by DOM, EPS, and mineral colloids—is one of the primary reasons that field-conditioned particles exhibit different metal-binding behavior compared to virgin laboratory polymers (Figure 5) [71,75].

The role of natural colloids intersects with DOM effects because iron oxides, humic colloids, and clay nanoparticles can attach to MPs and form composite particles. These composites behave as hybrid sorbents with metal affinities that differ from pristine MPs, often exhibiting stronger binding and altered desorption kinetics. Thus, the interpretation of MP effects on metal bioavailability requires measurement of not only polymer identity but also corona composition and colloid associations [75].

### 7.2. Competition and Co-Contaminants

Competitive binding among Cd, Zn, Cu, and Pb complicates both adsorption and toxicity. A stronger-binding ion may displace a weaker one from aged surfaces or biofilms, so multi-metal experiments often yield different results than single-metal studies, even when polymer type is held constant [15].

Evidence from mixed-contaminant systems also suggests that co-occurring sorbents modify those outcomes. For example, MPs may reduce sorption of a metal to another solid phase, thereby shifting the dissolved fraction and changing plant accessibility rather than simply adding new adsorption capacity [17,76]. Nutrients and organic co-contaminants matter because they alter competitive sorption, rhizosphere pH, and ligand supply. Phosphate is especially relevant in As-dominated systems, while nitrogen transformations can indirectly change pH and redox. Organic contaminants associated with MPs may also increase complexation and thereby change the mobility of PTEs bound at the particle–water interface [15,35]. Recent soil-speciation studies therefore caution against generalization across polymers or soils. Some MP types shift PTEs toward more acid-soluble fractions, whereas others favor less labile pools; the direction depends on the coating state, competing surfaces, and the extraction method used to define the operational fraction [54,77].

#### Co-Stressors and Multi-Contaminant Scenarios

Real agricultural soils rarely present MP–PTE interactions in isolation; fertilizer regimes, salinity, and co-occurring organic pollutants routinely modify the same competitive-sorption and rhizosphere processes discussed above, though this remains one of the more thinly evidenced areas of the plant–soil MP–PTE literature. Fertilizer-induced ionic strength is among the more tractable examples: ammonium- and phosphate-based fertilizers raise soil ionic strength and, as already noted for saline soils, can compress the electrical double layer around MP surfaces, favoring heteroaggregation and reducing electrostatic repulsion; phosphate addition also directly competes with arsenate for Fe-oxide sorption sites, meaning that fertilization practices can shift As mobility independently of and potentially in the same direction as MP-driven effects on the same sites [71,73]. Organic pollutant co-contamination is relevant mainly for the subset of PTEs, such as Cu and Hg, that engage substantially in organic complexation; DOM and co-occurring organic contaminants compete for many of the same complexation sites, so MP-associated organic coatings and independently derived organic pollutant loads are not easily separated as exposure sources once both are present in the same soil [75,78]. Salinity intersects with both pathways because it raises ionic strength while also altering plant osmotic status and root exudation, which, in turn, changes the ligand supplies available for metal complexation in the rhizosphere.

The main data gap is that almost none of the plant–soil MP–PTE studies reviewed above manipulates more than one co-stressor at a time; fertilizer, salinity, and organic-pollutant effects on MP–PTE interactions are therefore inferred here by combining separate single-stressor studies rather than drawn from studies that manipulated these factors jointly with MPs and PTEs in the same experimental system. Multi-factor pot or field experiments that cross MP treatment with fertilizer regime, salinity level, and organic pollutant load, ideally combined with a lability metric such as DGT, would directly test whether these co-stressor effects are additive or interactive with MP-driven PTE mobilization.

### 7.3. MP-Induced Modifications of Soil Physicochemical Properties

MPs affect PTE behavior not only by directly adsorbing metals but also by modifying the soil physicochemical environment that controls metal speciation, lability, and transport. This indirect pathway is essential because changes in soil pH, dissolved organic matter, ionic strength, aggregation, water retention, redox microsites and microbial activity can alter the chemical form and mobility of metal ions; even total PTE concentration remains unchanged [78].

#### 7.3.1. Soil Physical Structure Modifications

Bulk density and porosity: Experimental studies demonstrate that MP incorporation systematically alters soil physical structure. Degraded PE microplastics (1–5% *w*/*w*) reduced soil bulk density by 0.1–0.4 g cm^−3^ and increased porosity by creating water movement channels, enhancing evaporation and altering water dynamics in Albic Luvisol [79]. Polyester microfibers (0.1–0.4%) significantly enhanced pores > 30 μm while reducing pores < 30 μm, changing the pore-size distribution that governs metal diffusion pathways. These structural changes influence the contact frequency among PTEs, MP surfaces, clay minerals, Fe/Mn oxides, OM, and roots [80].

Aggregate stability: MP effects on aggregation are size- and concentration-dependent. Small MPs (<500 μm) participate in both small and large aggregate formation, but aged PS and PP MPs with oxygen-containing functional groups significantly increased aggregate disruption rates [81]. Conversely, Mondol et al. [64]) reported that 30% of PE macrofibers added to soils were incorporated in aggregates > 2 mm, though they reduced organic content of larger aggregates. In fine-textured soils, MPs can reduce cohesion between soil components, affecting water-stable aggregates that indicate resistance to erosion [64,79]. These aggregate dynamics directly control PTE accessibility: when MPs promote heteroaggregation with minerals and OM, MP-associated PTEs may become retained in less labile pools; when MPs disrupt aggregates or create preferential flow paths, particle-bound or colloid-bound PTEs may be transported toward root-active zones [79,81].

Water retention: High concentrations of polyethylene MPs (2%) altered soil texture and reduced water-holding capacity, while lower concentrations increased water retention through pore modification [64,79]. Reduced water-holding capacity decreases plant production and creates water-potential stress that affects root uptake of both water and dissolved PTEs [79].

#### 7.3.2. Soil Chemical Condition Modifications

pH changes: Weathered MPs, biodegradable polymers, and additive-containing plastics release dissolved organic compounds, oligomers, plastic additives, or degradation products that alter soil pH. In Cd-contaminated systems, PE microplastics increased soil acidity, enhancing Cd^2+^ mobility and root uptake in lettuce. Conversely, alkaline biochar–MP composites can raise pH, precipitating metals as carbonates or hydroxides [78].

Dissolved organic matter (DOM) composition: MPs modify DOM quality and quantity. Biodegradable microplastics (PLA and PBAT) release intermediate compounds that compete with naturally occurring soil DOC as microbial substrates, altering carbon cycling and the organic ligands available for metal complexation [64]. MPs can adsorb soil organic matter and act as binding agents for mineral particles, changing the DOM pool that controls metal speciation. These substances alter free ion activity; hydrolyzed species; carbonate complexes; organic complexes; colloidal associations; and precipitated fractions of Cd, Pb, Cu, Zn, As, and other PTEs [75,82].

Ionic strength and competitive effects: MP-associated DOM changes ionic strength and ligand availability. In marine-influenced systems, the humic acid concentration (0.1–5 mg/L) significantly altered trace metal (Cd, Cu, Pb, Zn, Ni, and Co) speciation and adsorption on PE and PP microplastics, with competitive effects among metal ions for limited binding sites. Similar competitive dynamics occur in soil pore water, where MP-released organic compounds may complex metals and change their partition coefficients [78]. Therefore, MPs can indirectly increase or decrease PTE adsorption by changing the chemical environment in which metal ions partition among pore water, soil solids, MP surfaces, and root interfaces. Moreover, MPs modify biological and rhizosphere processes. MP surfaces are rapidly colonized by biofilms and eco-corona composed of extracellular polymeric substances, humic substances, proteins, mineral colloids, and microbial cells. These coatings introduce carboxyl, hydroxyl, phosphate, amine, and sulfhydryl groups that can increase PTE adsorption. However, microbial respiration, root exudation, and biofilm metabolism can also generate microscale pH and redox gradients that promote desorption or transformation of PTEs. Thus, MP aging and biofilm formation may increase apparent adsorption capacity while still increasing PTE lability under rhizosphere conditions [83]. Overall, MPs should be interpreted as indirect modifiers of metal behavior, as well as possible sorbents or vectors.

## 8. Bioavailability and Crop-Safety Relevance: From Soil Pools to Plant Exposure

Microplastics alter bioavailability through two coupled routes: they change partitioning among soil pools and they modify the flux of PTEs to root surfaces. The critical distinction is not simply whether a metal is adsorbed to an MP but whether the adsorbed pool remains kinetically accessible under rhizosphere conditions [19,47].

**Figure 6:**
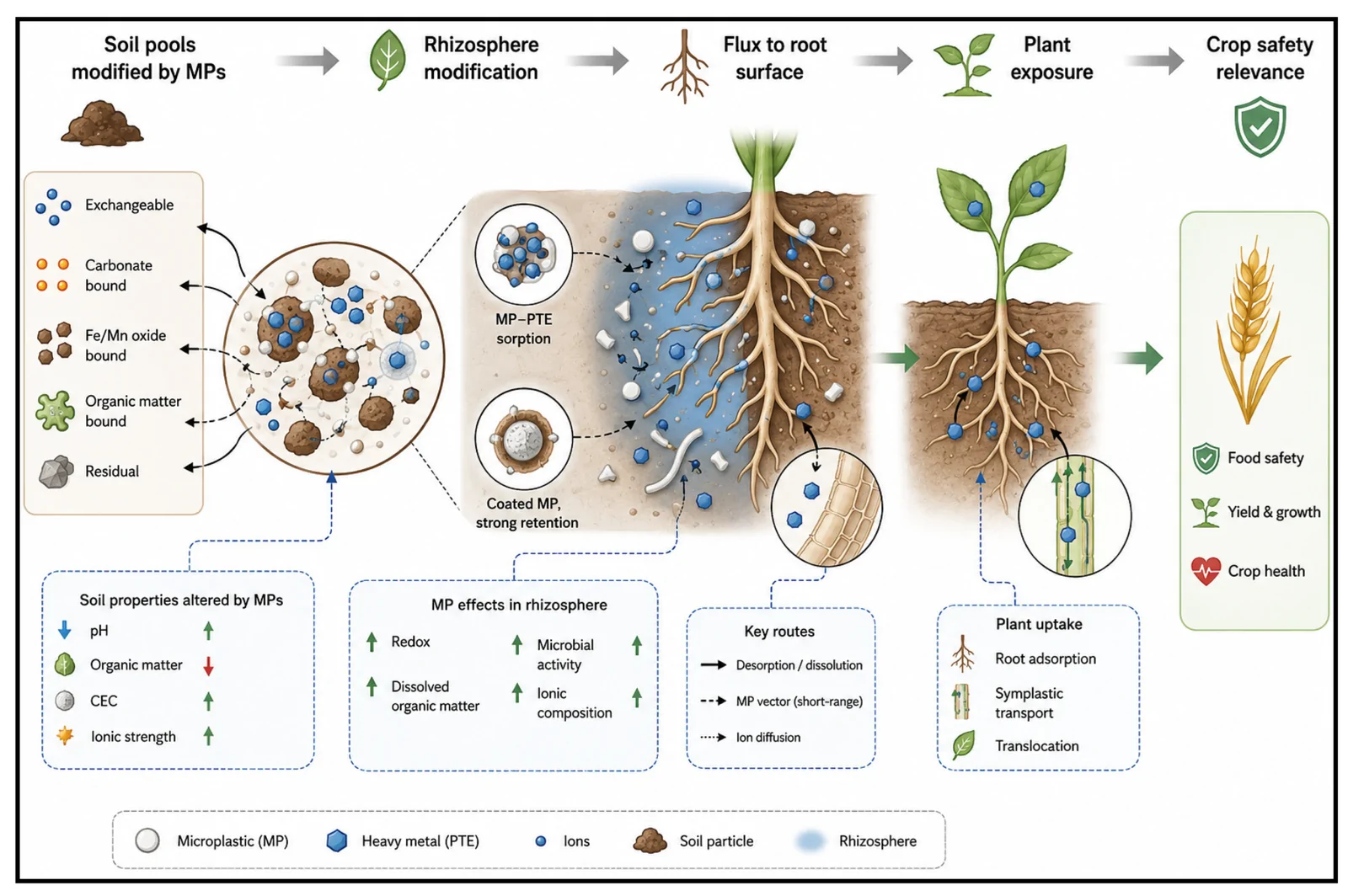
Conceptual framework linking MP-associated PTE partitioning, soil-property changes, rhizosphere processes, root-surface flux, and plant exposure, illustrating PTE bioavailability as a dynamic process with implications for crop safety. Arrow direction indicates progression from soil pools through rhizosphere processes to plant exposure; colors distinguish process domains and particle/contaminant classes. Created by the authors based on concepts reported in [13,19,34,47,84,85].

This point helps reconcile apparently contradictory results. When sorbed species are strongly retained within coated surfaces or stable heteroaggregates, MPs may reduce the labile pool. When sorption is reversible or particles move into root-active microsites, the same MPs can elevate plant exposure by acting as short-range vectors. These interacting processes are synthesized conceptually in Figure 6, which links changes in soil PTE pools and MP–PTE interactions with rhizosphere processes; root-surface flux, plant uptake, and ultimately crop-safety relevance [13,19,34,47,84,85].

Meta-analytic and experimental evidence indicates that smaller particles, low-organic-matter soils, and weaker competing sorbents often favor increased lability, whereas strong aggregation and coated surfaces may reduce it. This is why polymer type, by itself, rarely predicts plant uptake without accompanying information on soil texture, organic matter, and particle aging [84,85]. Operational measurement remains method-dependent. Exchangeable extractions can respond quickly to pH and ionic shifts, whereas resupply-based methods such as DGT better capture the sustained flux available to roots. The strongest studies therefore triangulate extraction data with pore-water chemistry and plant-tissue burdens rather than relying on a single metric [19,34].

Method choice matters because CaCl_2_ extractions, DTPA or EDTA chelation, sequential extraction, pore-water analysis, and DGT do not interrogate the same pool or time scale. Opposite conclusions can therefore arise when one study emphasizes immediately exchangeable ions whereas another emphasizes resupply or operational redistribution among fractions. For that reason, claims about increased or decreased bioavailability should be interpreted against the exact analytical definition that is used and, ideally, triangulated with plant-tissue data.

### 8.1. Crop-Safety Implication of MP-Mediated PTE Bioavailability

From a crop-safety perspective, the main concern is not the total concentration of PTEs in the soil but the fraction that remains soluble, exchangeable, or resuppliable to pore water during root uptake. MPs can modify this fraction by acting as sinks, vectors, or indirect reactivity modifiers. When MP-associated PTEs are retained within stable aggregates, mineral coatings, or biofilms, plant exposures may decrease. Conversely, when adsorption is reversible or when DOM, root exudates, acidic rhizosphere conditions, or particle transport promote desorption near root active zones, MPs may increase PTE uptake and phytotoxicity. Therefore, crop-safety assessment should integrate PTE lability, pore-water chemistry, rhizosphere processes, and edible tissue accumulation rather than relying only on total soil PTE concentrations.

### 8.2. Desorption Reversibility and Metal-Specific Bioavailability from MP Surfaces

Desorption behavior differs among PTEs. Cd and Zn are generally more labile than Pb and Cu because they are more readily exchanged from weak sorption sites and are strongly affected by pH and competing cations [86,87]. Pb is often less bioavailablebecause it is retained by phosphates, carbonates, Fe/Mn oxides, OM, and mineral coatings on MPs. Cu is strongly influenced by OM and biofilm-derived ligands, so its desorption depends heavily on DOM composition and microbial coatings. Ni usually shows moderate mobility and is sensitive to pH and oxide abundance. Cr bioavailability depends on the valence state, with Cr(VI) being more mobile and toxic than Cr(III), so MP effects are likely mediated through redox conditions and Fe/Mn oxide interactions. As differs from cationic metals because it occurs mainly as oxyanions and is controlled by Fe oxide binding, phosphate competition, and redox transformations. Hg is also distinct because its availability depends on organic binding, sulfide chemistry, biofilm activity, and microbial methylation rather than simple cation exchange.

Soil conditions determine whether MP-associated PTEsremain immobilized or become bioavailable. Acidic pH promotes desorption of Cd, Zn, and Ni by protonating reactive surface groups, reducing negative surface charge, and increasing competition between H^+^ and metal cations. Neutral to alkaline pH usually favors retention through deprotonated functional groups, cation exchange, carbonate association, or precipitation. Dissolved organic matter may either decrease free ion activity by complexing metals or increase mobility by forming soluble MP–DOM–PTE associations. Therefore, desorption from MPs should be interpreted together with soil pH, clay minerals, Fe/Mn oxides, OM, competing ions, redox state, and rhizosphere ligand release.

### 8.3. Rhizosphere Processes

The rhizosphere is the zone where many adsorption results are either amplified or reversed. Root exudates change pH, release organic ligands, and stimulate microbial activity, all of which can promote desorption from MP-associated pools or shift metals between dissolved, colloidal, and biofilm-bound forms [13,88].

Microbial colonization intensifies this effect because EPS-rich biofilms create new binding sites and local redox gradients. In aged systems, these biofilms can increase apparent adsorption capacity while simultaneously preserving a fraction that is exchangeable under rhizosphere ligands, which helps explain why stronger sorption does not always mean lower uptake [64].

Root-driven stabilization and disruption of aggregates occur simultaneously. Exudates may help retain MPs in microaggregates, while physical root growth may release them into pore channels. Consequently, plant species and rhizosphere traits become part of the exposure mechanism, not merely passive recipients of it [74,82].

**Figure 7:**
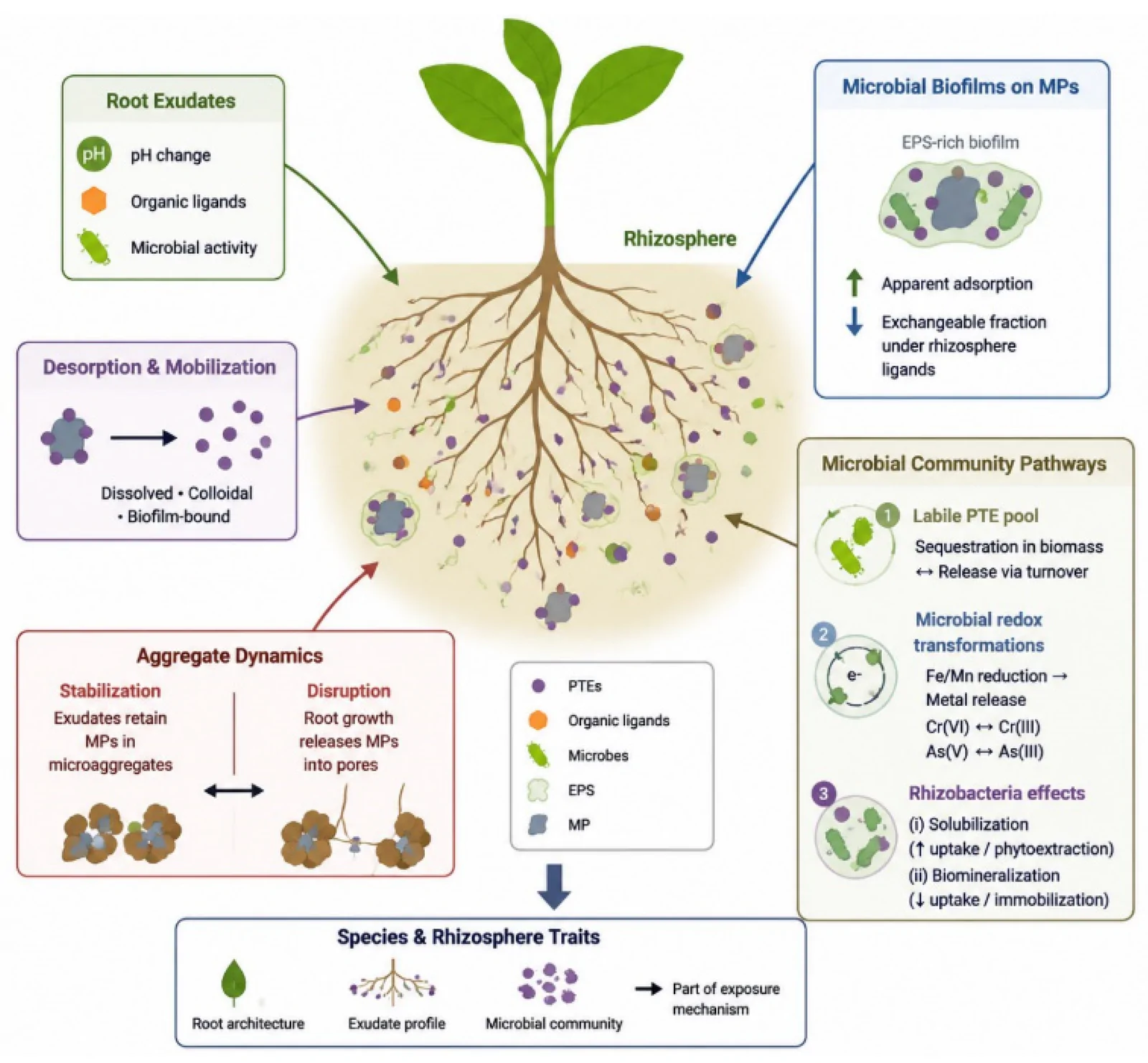
Conceptual framework illustrating rhizosphere-mediated transformation, mobilization, and stabilization pathways of contaminants associated with microplastics. The framework integrates root-exudate effects, microbial biofilm formation, aggregation–disaggregation dynamics, desorption processes, and microbial transformation pathways. Arrow direction indicates mobilization, stabilization, or transformation pathways, as labeled in the figure; colors distinguish the corresponding process domains.

The microbial community itself, rather than only the biofilm structures it forms on MP surfaces, is an active regulator of PTE bioavailability in the rhizosphere and warrants explicit treatment. Rhizosphere microbial taxa influence PTE speciation through at least three coupled pathways. First, microbial biomass and turnover directly control a labile PTE pool: microbial cells sequester PTEs intracellularly and on cell-surface functional groups, and this pool is subsequently released back into pore water as cells turn over, meaning the microbial community functions as a dynamic buffer rather than a one-way sink [89]. Second, microbially mediated redox transformations, distinct from the abiotic redox effects discussed in Section 7, directly determine the mobility of the redox-sensitive PTEs in this review: Fe- and Mn-reducing bacteria can dissolve the oxide phases that otherwise retain Cd, Pb, Cu, and Zn, releasing co-precipitated or adsorbed metals into solution, while microbial Cr(VI) reduction and As(V)/As(III) interconversion are substantially biotic rather than purely geochemical processes [90]. Third, plant growth-promoting and metal-resistant rhizobacteria can actively modulate plant metal uptake, either by producing siderophores and organic acids that increase PTE solubility and, in some reported systems, phytoextraction, or by biomineralizing and immobilizing PTEs extracellularly, thereby protecting the plant from excess uptake; which of these two outcomes dominates depends on the specific microbial taxa present, PTE identity, and the degree to which MP-associated biofilms have already altered the structure of the local microbial community. Because MP surfaces are preferentially colonized relative to bulk soil and can therefore act as a selective habitat for particular microbial functional groups [91], MP presence has the potential to shift the balance among these three pathways in ways that are not yet well resolved in the plant–soil literature reviewed here; this is flagged explicitly as a priority direction in Section 12. A conceptual framework was developed to integrate the major biological, chemical, and physicochemical processes controlling contaminant behavior in the rhizosphere environment (Figure 7).

### 8.4. Plant Uptake Routes

Plant uptake routes remain governed by root-surface adsorption, apoplastic movement, and transporter-mediated symplastic uptake, but MPs can modify each step indirectly through changes in pore-water chemistry or directly through localized particle contact at the root interface. Evidence for internalization is stronger for nano-sized plastics than for larger MPs, so most plant–soil interpretations still rest primarily on chemistry-mediated exposure rather than on bulk particle uptake [92].

Microplastics influence uptake through changed external speciation, altered transporter competition, and rhizosphere-mediated redistribution of PTEs. In rice and lettuce systems, the clearest responses occur where MPs shift pH, DOC, or microbial activity sufficiently to change the bioavailable pool reaching the root surface [18].

Translocation to shoots depends on xylem loading and sequestration within root tissues, so increased root exposure does not automatically translate to edible-tissue accumulation. Even so, several recent plant studies show that MP-induced changes in rhizosphere chemistry can increase aboveground accumulation of Cd or As under some exposure regimes, which is why uptake and translocation must be interpreted together [13,18].

## 9. Phytotoxicity Under Co-Exposure to Microplastics and Potentially Toxic Elements

Phytotoxicity under combined MP–PTE exposure should be interpreted as the biological endpoint of the processes reviewed above. The most informative studies are those that connect soil or rhizosphere chemistry to plant responses rather than treating biomass effects as isolated symptoms. Across current evidence, outcomes are clearly non-uniform: synergistic, additive, and antagonistic interactions all occur depending on polymer type, particle dose, weathering state, PTE identity, and plant physiology [13,93].

Quantitative examples illustrate this variability (Table 5). In lettuce grown in Cd-contaminated soils, 10% PE MPs reduced biomass by 16.8–29.3% and increased plant Cd accumulation, whereas lower PE additions had weaker effects [18]. In rice exposed to As (III), PS or PTFE at 0.2 g L^−1^ intensified toxicity, with maximum root biomass reductions of 21.4–25.4%, but lower particle additions partly attenuated the As stress response [56]. By contrast, some maize systems exposed to polystyrene nanoplastics exhibit antagonistic behavior, showing that stronger particle presence does not always translate into stronger metal toxicity [77].

These differences indicate that phytotoxicity is linked to bioavailability but not by a simple one-to-one relation. MPs can change nutrient competition, microbial mediation, and stress signaling at the same time that they change the labile PTE pool, so physiological injury should be interpreted as the product of co-varying chemical and biological mechanisms.

### 9.1. Biomarker-Specific Responses Under MP–PTE Co-Exposure

Studies have shown that MP-PTE co-exposure alters plant performance through several biomarker categories, but the strength of evidence differs among PTEs (Table 6). The most frequently reported are seed germination, root elongation, biomass production, chlorophyll contents, photosynthesis efficiency, oxidative stress, antioxidant enzyme activity, nutrient imbalance, metal accumulation, and genotoxic or stress signaling responses [97]. Cd and As are the best supported cases. Cd toxicity often increases when MPs increase Cd lability, pore-water Cd, or root-zone Cd delivery [18]. Reported responses include reduced root and shoot biomass; inhibited root elongation; increased Cd accumulation in roots and shoots; decreased chlorophyll contents; elevated reactive oxygen species (ROS); increased malondialdehyde accumulation; and altered antioxidant enzyme activities such as SOD, CAT, POD, and APX [18]. These responses are particularly evident when MPs acidify soil, disrupt aggregates, increase DOM, or transport labile Cd into the rhizosphere. However, antagonistic or weak responses may occur when aged or mineral-coated MPs immobilize Cd in less labile pools. As responses are controlled mainly by redox state, phosphate competition, Fe plaque and rice rhizosphere chemistry; low MP doses may reduce stress, while higher doses may intensify toxicity.

For Pb, effects are usually limited unless MPs promote particle-bound transport, acidification, or ligand-driven mobilization. Cu and Zn responses are complex because they are nutrients at low levels but toxic when labile concentrations rise. Ni and Cr evidence is also limited; Ni effects depend on pH and oxide-bound pools, while Cr must be interpreted by valence state, especially Cr(VI) versus Cr(III). Hg is the least resolved because its toxicity depends on organic binding, sulfides, biofilms, and microbial methylation. Overall, MP–PTE phytotoxicity is not uniform. Biomarker responses depend on PTE chemistry, MP effects on labile metal pools, soil conditions, and plant growth stage [97].

### 9.2. Oxidative Stress and Cellular Damage

Oxidative stress is one of the most consistently reported pathways because combined MP–PTE exposure can elevate reactive oxygen species, membrane damage, and antioxidant demand. In rice and lettuce systems, increased O^2−^ and H_2_O_2_ accumulation, lipid peroxidation, and shifts in enzymatic defense have been reported when MPs intensify the effective exposure to As or Cd [18,56].

The direction of antioxidant-enzyme responses is not uniform across studies, which is, itself, informative. Moderate co-exposure may transiently increase SOD, CAT, POD, or glutathione-related activity as a defense response, whereas stronger or prolonged exposure can overwhelm that compensation and coincide with biomass loss, chlorophyll decline, and membrane injury. The review therefore interprets enzyme shifts in relation to dose and accompanying uptake data rather than as stand-alone indicators [13,48,56].

### 9.3. Photosynthesis and Metabolic Disruption

Photosynthetic impairment reflects both chemical toxicity and altered plant metabolism. Reported effects include lower chlorophyll content, reduced fluorescence performance, suppressed RuBisCO-related activity, and disturbed nutrient balance, especially where MPs increase the bioavailable fraction of Cd or amplify As-related oxidative stress [18,56]. Metabolic changes such as altered amino-acid pools, organic-acid exudation, and carbon–nitrogen allocation are also relevant because they provide feedback on rhizosphere chemistry. Once metabolism changes, the plant can modify the very ligand environment that controls desorption and uptake, producing non-linear responses over time.

### 9.4. Genotoxicity and Stress Signaling

Evidence for genotoxicity and stress signaling is still thinner than for oxidative injury, but available studies suggest that combined exposure can enhance damage signaling when bioavailable PTE fluxes remain high, particularly in Cd- and As-based plant exposure systems where MPs increase effective metal exposure, ROS production, lipid peroxidation, and antioxidant-enzyme responses [58]. At present, this is best treated as an emerging subfield rather than a firmly resolved endpoint. For that reason, the review emphasizes a hierarchy of confidence: biomass, uptake, and oxidative-stress responses are already well supported; photosynthetic and metabolic disruption are moderately supported; and genotoxicity mechanisms require more harmonized plant–soil studies that link transcript or signaling data to measured lability and accumulation.

## 10. Methodological Considerations for Interpretation of MP–PTE Interactions

Methodological discussion is retained only because it determines how adsorption, mobility, and bioavailability are inferred. Batch sorption tests identify mechanisms, soil incubations track fraction shifts, column studies reveal mobility, and pot trials connect those changes to plant response. None of these designs alone is sufficient, which is why conflicting conclusions often arise across the literature [17,34].

The strongest evidence comes from studies that combine realistic particle characterization, controlled soil description, and at least one lability metric with plant data. In contrast, studies that use pristine particles at very high doses without reporting weathering state or soil context are best interpreted as process-identification experiments rather than direct risk evidence.

Metrics such as pore-water concentration, DGT, selective extraction, and sequential fractionation do not interrogate identical pools. Their disagreement is therefore a feature to be interpreted, not merely a methodological flaw. This section is kept concise because its role is to support interpretation of the central processes reviewed above.

Best practice now points toward triangulation: spectroscopic identification of polymers, explicit reporting of size and weathering, multiple indicators of PTE lability, and plant uptake endpoints interpreted together (Table 7). That combination makes synthesis more robust and reduces the disconnected, highly general reading that can arise when methods are described without their interpretive role.

### 10.1. Detection and Characterization Tools

Polymer identification commonly relies on FTIR and Raman spectroscopy, while particle size and morphology are resolved through microscopy and imaging workflows. These tools matter because polymer verification, size distribution, and weathering state directly influence how adsorption and phytotoxicity results should be compared across studies.

Metal quantification usually relies on ICP-based methods after digestion, with DGT, pore-water analysis, or selective extraction used to approximate plant-accessible pools. Contamination control during MP analysis is equally important because false positives in fiber identification can distort both occurrence and co-exposure interpretation [26].

### 10.2. Data Harmonization and Reporting Standards

Data harmonization is essential because many apparent contradictions in the literature stem from poor comparability rather than from genuinely opposite mechanisms. Polymer type, particle size, aging, soil texture, pH, organic matter, background contamination, and exposure duration should all be reported as minimum metadata for interpreting MP–PTE interactions. Plant reporting should likewise include growth stage, tissue-cleaning procedure, translocation metrics, and whether the study addresses dissolved uptake, particle contact, or both. Without such detail, it becomes difficult to distinguish genuine changes in bioavailability from artifacts of extraction, exposure design, or tissue contamination.

Standardized reporting does not replace mechanistic analysis, but it makes synthesis defensible. This is why methodological detail is treated here as a support for interpretation rather than as a separate review theme.

### 10.3. Rubric for Evidence-Strength Labels Used Throughout This Review

The “strong/moderate/limited” evidence-status labels used in Table 3 and the “strong/moderate/limited” evidence-strength labels used in Table 6 follow the same three-part rubric, applied qualitatively across the reviewed literature rather than through a formal quantitative scoring system: (i) approximate number of plant–soil (not aquatic-only) studies directly addressing the PTE–MP combination, following the same relative-density approach outlined in Section 4.7; (ii) whether reported effects are corroborated across more than one crop species, MP polymer type, or independent research group, as opposed to resting on a single study; and (iii) whether at least one lability or speciation metric (DGT, sequential extraction, or pore-water analysis) accompanies the reported effect, rather than total-concentration or biomass data alone. A label of “strong” requires a plant–soil (not aquatic-only) evidence base with multi-study or multi-species corroboration and at least partial lability-metric support; “moderate” indicates plant–soil evidence exists but is corroborated across fewer independent systems or relies more heavily on total-concentration data; and “limited” indicates that plant–soil evidence for that specific PTE is sparse, mechanistically inferred by analogy with better-studied PTEs, or drawn primarily from a small number of studies. We report this rubric explicitly here so that the qualitative labels used in Table 3 and Table 6 can be checked against the reviewed literature rather than treated as an unstated judgment call.

### 10.4. Limitations of the Available Evidence Base

Beyond the rubric above, several structural limitations affect the evidence synthesized in this review and should condition how strongly its conclusions are generalized. First, the large majority of the plant–soil literature reviewed here is derived from controlled laboratory pot or greenhouse experiments rather than field trials; field-scale, multi-season studies that connect MP measurements, labile PTE indicators, and crop tissue burdens under real agricultural management remain rare (Section 12). Second, many experimental studies use MP concentrations (frequently in the 0.1–10% *w*/*w* range) that are one to several orders of magnitude higher than concentrations reported in agricultural soil surveys, so dose-response relationships characterized under high-dose laboratory conditions may not extrapolate linearly or even in the same direction to field-relevant exposure levels. Third, long-term or multi-year studies that capture realistic aging, weathering, and biofilm succession under field conditions are scarce relative to the number of short-duration batch or single-season pot studies; most aging-related conclusions in this review therefore rest on accelerated laboratory aging protocols (e.g., UV chambers or controlled wet–dry cycling) whose rate and endpoint may not fully represent field aging trajectories. Fourth, as already noted in the unnumbered subsection “Co-Stressors and Multi-Contaminant Scenarios”, co-stressor scenarios involving fertilizer regimes, salinity, and organic-pollutant co-contamination are addressed by this review mainly through separate single-stressor studies rather than through studies that manipulated MPs jointly with these factors. These limitations do not invalidate the mechanistic patterns discussed throughout this review, but they do mean that quantitative effect sizes (e.g., the biomass reduction ranges reported in Section 9) should be interpreted as illustrative of reported laboratory-scale effects rather than as field-validated dose-response estimates.

## 11. Mechanistic Interpretation of MP-Mediated PTE Behavior in Plant–Soil Systems

This section integrates, rather than restates, the scale-specific evidence developed in Section 5 (particle-surface mechanisms), Section 7 (soil-system redistribution), and Section 8 (bioavailability and plant exposure). Where a controlling variable such as aggregation, pH, or aging appears to support conflicting conclusions across those sections—for example, aggregation reducing PTE mobility in some reported systems while enhancing it in others—this is treated here explicitly as evidence of context-dependence (soil texture, aggregate stability, and MP loading) rather than as an unresolved contradiction, and the conditions favoring each outcome are stated directly below.

MP–PTE interactions in plant–soil systems are controlled by a chain of linked processes: particle conditioning sets the reactive interface; soil minerals and DOM regulate competition and aggregation; rhizosphere processes shift desorption and resupply; and plant physiology determines the final uptake and injury outcome. The literature supports this chain much better than a simple binary of ‘adsorption equals immobilization’ or ‘transport equals toxicity’ [13,15,39].

At the plant-response layer, uptake, oxidative injury, photosynthetic disruption, and altered nutrient balance represent integrated consequences of those upstream processes. The current evidence base is strongest for Cd and As systems, which should be acknowledged explicitly when generalizing across all PTEs.

Vector-dominated outcomes are most likely when particles are small, conditioned, present in root-active soil, and associated with weakly buffered matrices or strong ligand-driven desorption. Sink-dominated outcomes are more likely when coatings and heteroaggregates immobilize PTEs within less exchangeable pools. Many real systems move between these states rather than remaining fixed in one mode.

The conceptual model is therefore a synthesis of the reviewed evidence rather than a stand-alone schematic: it explains when adsorption, mobility, bioavailability, and phytotoxicity converge and when they diverge under different boundary conditions.

## 12. Knowledge Gaps and Future Research Directions

Several knowledge gaps limit confident prediction of MP-mediated metal behavior under field conditions. Realistic MP concentrations in agricultural soils are often lower than doses used in pot experiments, yet MPs may be highly heterogeneous in space and concentrated in surface layers, making exposure episodic and patchy. Long-term aging in soils, including repeated wetting–drying and biofilm succession, is under-represented in short-duration experiments despite its strong influence on surface reactivity and desorption kinetics.

Field validations that connect MP measurements, labile metal indicators, and crop tissue burdens remain limited. Co-contaminant mixtures are the rule in real soils, including multiple metals, nutrients, pesticides, and organic pollutants, yet many mechanistic studies use single-metal systems, limiting inference about competitive binding and selective release. Studies that explicitly incorporate multi-metal competition and co-contaminant chemistry will better represent agricultural exposure scenarios. Future research should prioritize field-scale and long-term studies using environmentally realistic MP concentration, naturally aged particles, representative soil types, and crop-relevant exposure scenarios.

Standardized bioavailability metrics are still evolving. Comparative use of DGT, selective extraction, and kinetic resupply approaches could clarify discrepancies across studies, especially when combined with rhizosphere microprofiling and characterization of corona composition. Crop-specific responses and cultivar differences are also underexplored, including variations in exudate chemistry and transporter expression that can shift both uptake and rhizosphere-driven desorption from MPs.

Mechanistic tracing of metal transfer from MP surfaces to roots is a priority. Isotopic labeling, surface-sensitive spectroscopy, and imaging approaches could establish whether increased uptake originates from MP-delivered metal, from MP-induced mobilization of native-soil metal pools, or from physiological changes in plants that alter uptake capacity. Such tracing would strengthen causal interpretation and support predictive models for risk assessment.

Beyond these process-level priorities, several emerging research directions could substantially advance the field. Omics techniques, including rhizosphere metagenomics, metatranscriptomics, and root or leaf metabolomics, could resolve the microbial and plant physiological mechanisms discussed in Section 8.3 at a resolution not accessible through bulk chemical measurements alone, directly addressing the microbial-mediation questions flagged above as unresolved. Multi-stress experimental designs that combine MP–PTE co-exposure with drought, salinity, or engineered nanoparticle co-contamination would extend the co-stressor synthesis in the unnumbered subsection “Co-Stressors and Multi-Contaminant Scenarios” from a literature-inferred discussion to directly tested interactions, which is particularly relevant, given that salinity and drought are already common co-occurring stressors in the agricultural soils where MP–PTE contamination is most reported. Predictive modeling approaches, including machine learning-based prediction of MP–PTE sorption behavior from polymer and soil descriptors, and process-based models that couple MP transport with PTE speciation, could help generalize the conditional sink/vector/modifier framework proposed in this review (Table 1) beyond the specific systems in which it was derived. Finally, more integrative environmental risk approaches that combine labile-PTE indicators, MP characterization, and crop tissue burden into a single risk index rather than evaluating MP and PTE risk separately would translate the mechanistic synthesis in this review into a form more directly usable for agricultural risk assessment and management (Section 13).

## 13. Mitigation and Management Options

Mitigation strategies should target both MP inputs and the conditions that convert sorbed PTEs into plant-accessible pools. Practical priorities include reducing residue persistence from mulching systems, improving control of sludge- and compost-borne plastics, and minimizing traffic-derived inputs to cultivated margins.

Management of PTE lability remains equally important. Amendments such as biochar, mineral sorbents, and pH-correcting materials may reduce bioavailability, but their performance should be evaluated in the presence of MPs because coating formation, DOM release, and aggregate change can modify the expected immobilization outcome.

Crop and microbiome management also matter. Selecting cultivars with lower translocation capacity, preserving beneficial mycorrhizal interactions, and monitoring rhizosphere conditions can help reduce plant exposure under co-contamination. These options are more realistic than assuming that MP removal from field soil is feasible once residues are widely dispersed.

At the governance level, the most defensible approach is integrated monitoring of MP inputs, labile PTE fractions, and crop tissue burdens rather than reliance on total metal concentration alone. That approach follows directly from the evidence synthesized in this review.

## 14. Conclusions

Microplastics (MPs) influence potentially toxic element (PTE) behavior in plant–soil systems through linked adsorption, aggregation, transport, desorption, soil-property modification, and rhizosphere mediation. Their effects are not uniform. Depending on polymer type, particle state, soil geochemistry, and rhizosphere conditions, MPs may reduce PTE availabilityby promoting sequestration or increase exposure by facilitating desorption, colloidal transport, and delivery to root-active zones.

The reviewed evidence from the literature shows that total soil PTE concentration alone is insufficient for evaluating plant exposure or crop safety risk. More informative assessment requires attention to labile fractions, pore-water chemistry, desorption reversibility, rhizosphere conditions, and metal accumulation in plant tissues. The strongest available evidence currently concerns Cd and As systems, while Pb, Cu, Zn, Ni, Cr, and Hg require more targeted plant–soil studies under realistic exposure conditions.

Overall, MPs should be treated as conditional reactivity modifiers rather than as universally acting sinks or vectors. Future progress depends on field-relevant dosing, aged particles, clearer reporting of polymer state and soil context, and tighter linkage between lability metrics and plant response. Under those conditions, the field will be better positioned to move from broad generalization toward predictive understanding of when MP–PTE co-contamination poses meaningful plant–soil risk.

## Figures and Tables

**Table 2:** Major sources of microplastics in soils and their relevance to MP–PTE interactions.

Input Pathway	Typical MP Forms	Common Polymers	Metal Linkage Pathway	Key Interaction Implications	Ref.
Plastic mulching and greenhouse films	Films, fragments	PE, PP, biodegradable blends	Co-occurrence with agrochemical metals; soil contact promotes aging	Fragmentation increases surface area and creates reactive sites that can bind metals; rhizosphere exposure is direct	[31,32,33]
Biosolids and sewage sludge	Fibers, fragments	PET, PE, PP, PA	Sludge conditioning and organic coatings concentrate metals on MPs	Wastewater-conditioned MPs can show elevated adsorption potential and deliver metals with organic coronas	[34]
Wastewater irrigation and runoff	Fibers, fragments	Mixed	Transport of both MPs and dissolved/particulate metals	Co-transport increases contact frequency and can shift metal partitioning in the pore-water phase	[5]
Atmospheric deposition and roadside inputs	Fibers, fragments	PET, PP, rubbery polymers	Deposition can co-deliver traffic-related metals	Fine fibers can enter aggregates and interact with DOM, influencing mobility and lability	[35]
Littering and industrial losses	Pellets, fragments	PE, PP, PS	Pigments, stabilizers, and co-residues may contain metals	Additive chemistry can introduce ligands or change charge, modifying sorption behavior	[36]

**Table 3:** Priority potentially toxic elements selected for this review and relevant interaction controls in plant–soil MP systems.

PTE	Evidence Status in MP–Plant–Soil Literature	Dominant Soil Speciation Controls	Typical Mobility Pattern	Plant Exposure Considerations	MP-Sensitive Mechanisms	Ref.
Cd	Strong evidence base; frequently used PTE	pH, oxide surfaces, Fe/Mn oxides, OM binding, and competing cations	Moderate to high mobility in acidic soils	Transporters shared with Zn/Fe; sensitive to rhizosphere ligands	Strong response to MP-driven pH/DOM shifts and to aged surface complexation	[41]
Pb	Moderate evidence base	Oxide, phosphate, and carbonate binding; low solubility except under acidification	Low mobility in most soils; locally mobile under rhizosphere acidification or particle-bound transport	Availability depends on localized pH change and particle-bound delivery rather than bulk soil concentration	MP surfaces provide alternative sorption sites (carboxyl and hydroxyl) that compete with soil oxides for Pb; particle-bound transport via preferential flow; rhizosphere acidification from MP degradation products solubilizes carbonate/phosphate-bound Pb	[14]
Cu	Moderate evidence base	Strong OM complexation and sulfide under reducing zones	Low dissolved fraction in OM-rich soils	Micronutrient and toxicant; redox and DOM mediate bioavailability	MP coatings and biofilms can change Cu partitioning substantially; competitive sorption	[42]
Zn	Moderate evidence base	pH, carbonates, exchange sites, organic matter, and competing cations	Higher lability than Cu in many soils	Competition with Cd; high sensitivity to labile pool changes	MP effects often track ionic strength and competitive sorption	[43]
As	Strong evidence base, especially in rice	Fe–oxide binding, phosphate competition, and redox	Oxyanion mobility increases under reducing conditions	Uptake via phosphate transporters; speciation critical	MP effects tend to be indirect via redox and microbial shifts	[35]
Cr	Limited but important redox-sensitive case	Cr(III)/Cr(VI) speciation, redox potential, Fe/Mn oxides, and organic matter	Cr(VI) more mobile and toxic; Cr(III) less mobile	Uptake and toxicity depend strongly on valence state	MP-driven Cr(VI)/Cr(III) redox transformation; MP surfaces provide electron-transfer sites for Cr(VI) reduction; plastisphere microbial communities alter Cr methylation/demethylation; competitive sorption on aged MP surfaces mobilizes Cr from Fe/Mn oxides	[40]
Ni	Limited but mechanistically relevant evidence	pH, Fe/Mn oxides, clay minerals, and organic matter	Moderate mobility depending on pH and oxide abundance	Can affect root growth and nutrient balance at elevated availability	MP-induced pH shifts alter Ni solubility; competitive sorption on MP carboxyl/hydroxyl groups displaces Ni from soil-exchange sites; MP biofilm coatings change Ni partitioning between dissolved and particulate phases	[41]
Hg	Limited but mechanistically important	Organic matter binding, sulfides, methylation, and microbial activity	Strongly form-dependent; methylated forms are highly bioavailable	Plant exposure depends on form and microbial transformation	MP effects likely mediated by biofilms, organic coatings, and microbial methylation pathways	[42]

**Table 5:** Representative quantitative outcomes of MP–PTE co-exposure with respect to plant biomass and PTE bioavailability direction.

Direction of MP Effect	Crop	PTE	MP Type/Dose	Quantitative Range to Report	Interpretation	Ref.
Toxicity enhanced	Leafy vegetables, e.g., lettuce	Cd	PE at a high dose such as 10%	Biomass reduction: 16.8–29.3%	MP addition increased Cd bioavailability and plant Cd accumulation	[18]
Toxicity enhanced	Rice	As (III)	PS or PTFE, 0.2g L^−1^	Root biomass reduction: 21.4–25.4%; leaf biomass reduction: 10.2–11.8%	Higher MP dose intensified As toxicity	[94,95]
Toxicity attenuated/biphasic	Rice	As(III)	Low dose of PS/PTFE	Partially alleviated	Low MP dose partly reduced As stress, showing non-linear response	[95]
Bioavailability increased	Lettuce/maize/wheat	Cd, Pb, Cu, or Zn, depending on the study	PE, PS, PP, and aged MPs		Increased lability linked to pH change, DOM release, desorption, or particle transport	[18,84]
Bioavailability decreased	Soil-plant system with stronger aggregation/coating	Cd, Pb, Cu, and Zn	Aged/coated MPs or aggregate-bound MPs		MPs acted as sinks by immobilizing PTEs in less labile pools	[96]

**Table 6:** MP-mediated effects on PTE bioavailability, plant biomarkers, and phytotoxic outcomes.

PTE	Strength of Available Evidence	Dominant Controls on Bioavailability	Commonly Affected Plant Biomarkers	Typical MP-Mediated Outcome	Ref.
Cd	Strong	pH, DOM, exchangeable Cd, and root-zone delivery	Biomass, root elongation, MDA, ROS, SOD, CAT, POD, chlorophyll, and Cd accumulation	Often intensified toxicity when Cd lability increases	[98]
As	Strong	Redox, Fe oxides, phosphate competition, and rice rhizosphere	Root/shoot biomass, chlorophyll, ROS, antioxidant enzymes, and As uptake	Dose-dependent; low MP may attenuate, while high MP may intensify	[56]
Pb	Moderate	Oxide/phosphate/carbonate binding and particle transport	Root inhibition, biomass, and oxidative stress	Limited unless Pb is mobilized	[99]
Cu	Moderate	DOM, OM, and biofilms	Chlorophyll, photosynthesis, oxidative stress, and nutrient balance	Variable; nutrient toxicity threshold-dependent	[100]
Zn	Limited	pH, exchange sites, and Cd competition	Growth, chlorophyll, nutrient balance, and oxidative stress	Variable; linked to micronutrient imbalance	[100]
Ni	Limited	pH, oxides, and exchangeable pools	Root growth, chlorosis, ROS, and nutrient uptake	Limited; likely pH/oxide-dependent	[17]
Cr	Limited	Cr(VI)/Cr(III), redox, and Fe/Mn oxides	Root inhibition, oxidative stress, and genotoxicity	Valence-dependent	[17]
Hg	Limited	Organic-matter binding, sulfides, and microbial methylation	Root/shoot biomass, oxidative stress, antioxidant enzyme activity (inferred from limited plant–soil evidence)	Least resolved of the priority PTEs; toxicity depends on organic binding, sulfide chemistry, biofilms, and microbial methylation rather than simple exchange	[18]

MP = microplastic; DOM = dissolved organic matter; MDA = malondialdehyde; ROS = reactive oxygen species; SOD = superoxide dismutase; CAT = catalase; POD = peroxidase.

**Table 7:** Core experimental designs and tools used to study MP–PTE interactions in plant–soil systems.

Study Design	Primary Question	Key Outputs	Strength	Common Limitation	Ref.
Batch sorption (single phase)	How do MPs bind specific metal species?	Isotherms, kinetics, and surface spectra	Mechanistic clarity under controlled chemistry	Ignores soil competition and corona formation	[17]
Soil incubation (speciation)	Do MPs shift metal fractions and soil chemistry?	Sequential fractions, pore water, and microbial indicators	Captures soil-mediated transformations	Extraction-based bioavailability proxies vary by protocol	[68]
Column transport	Can MPs transport metals through pores?	Breakthrough curves and aggregation metrics	Reveals mobility and heteroaggregation	Boundary conditions can differ from field structure	[59]
Pot/greenhouse trials	Does co-exposure change uptake and toxicity?	Tissue metal burdens, growth, and physiology	Links chemistry to plant outcomes	MP doses and aging can be unrealistic without field calibration	[64]
Field surveys	Do co-occurrence patterns align with uptake risk?	MP abundance, soil metals, and crop tissues	High relevance to management	Causality is hard to infer without mechanistic linkage	[59]

## Data Availability

No new data were created or analyzed in this study. Data sharing is not applicable to this article.
